# Unveiling the Oral Lesions, Dysgeusia and Osteonecrosis Related to COVID-19: A Comprehensive Systematic Review

**DOI:** 10.3390/jcm14041267

**Published:** 2025-02-14

**Authors:** Angelo Aliberti, Roberta Gasparro, Martina Mignogna, Federica Canfora, Gianrico Spagnuolo, Gilberto Sammartino, Noemi Coppola

**Affiliations:** 1 Department of Neurosciences, Reproductive Sciences and Odontostomatological Sciences, University of Naples Federico II, 80131 Naples, Italy; ange.aliberti@studenti.unina.it (A.A.); roberta.gasparro@unina.it (R.G.); martymig87@gmail.com (M.M.); federica.canfora@unina.it (F.C.); gspagnuo@unina.it (G.S.); noemi.coppola91@gmail.com (N.C.); 2 Therapeutic Dentistry Department, Institute for Dentistry, Sechenov University, Moscow 119991, Russia

**Keywords:** COVID-19, dysgeusia, mucormycosis, oral lesion, oral manifestations, osteonecrosis, systematic review

## Abstract

**Background/Objectives**: The oral cavity has garnered increasing attention as a site for viral infection and related pathological manifestations in coronavirus disease-19. This article aims to provide a comprehensive overview of SARS-CoV-2 (severe acute respiratory syndrome coronavirus 2)-related oral manifestations, including taste disturbances, oral lesions and osteonecrosis. **Methods**: A search was conducted up to September 2024 according to PRISMA (Preferred Reporting Items for Systematic Reviews) guidelines using the databases PubMed and Scopus. All the observational, case-series, case-report and cross-sectional studies written in English on oral manifestations related to COVID-19 disease and long-COVID disease were included. All other types of studies and studies based on oral manifestation after COVID-19 vaccination and oral impairment due to lockdown were excluded. The risk of bias of included studies was assessed using the Joanna Briggs Appraisal checklist. **Results**: A total of 104 articles including 23 case-report, 15 case-series, 8 case-control, 18 cohort and 40 cross-sectional studies were selected. The results showed that patients with COVID-19 were found to have a significantly higher prevalence of xerostomia (45–74%) and dysgeusia (32–59%) compared to non-infected individuals. Regarding oral mucosal lesions, ulcers, candidiasis and herpes simplex infections were frequently observed. As for osteonecrosis, a significant number of patients with COVID-19-associated rhinomaxillary mucormycosis presented with maxillary osteonecrosis due to fungal infection, primarily mucormycosis. The methodological quality of most of the studies was moderate/high. **Conclusions**: COVID-19 has been associated with a range of oral manifestations. The complex interplay of viral infection, immune response, medication use and stress likely contributes to these oral complications. Early recognition and management of these oral manifestations are crucial for improving patient outcomes and developing targeted preventive and therapeutic strategies for COVID-19-related oral health issues.

## 1. Introduction

Coronavirus disease 2019 (COVID-19) is an acute respiratory illness caused by the severe acute respiratory syndrome coronavirus 2 (SARS-CoV-2), a novel coronavirus first identified in December 2019 in Wuhan, China. SARS-CoV-2 is highly transmissible, facilitating rapid global spread and culminating in the declaration of a pandemic by the World Health Organization (WHO) on 11 March 2020 [1].

SARS-CoV-2 is transmitted primarily through respiratory droplets and aerosols, particularly in close-contact settings. However, direct contact with contaminated surfaces and fecal–oral transmission surfaces have also been implicated in the spread of the virus, albeit less prominently [2]. Upon entry into the human host, SARS-CoV-2 gains cellular access via its spike (S) glycoprotein, which binds to the angiotensin-converting enzyme 2 (ACE2) receptor, predominantly expressed on epithelial cells of the respiratory tract, gastrointestinal tract and vascular endothelium [3].

Clinical manifestations of COVID-19 infection range from asymptomatic and mild upper respiratory symptoms to severe pneumonia, acute respiratory distress syndrome (ARDS) and multi-organ failure [1,3]. Although respiratory involvement is the main one, mounting evidence indicates that SARS-CoV-2 has multisystemic effects, including gastrointestinal, cardiovascular, neurological and dermatological complications [4]. Notably, the oral cavity has garnered increasing attention as a site for both viral infection and related pathological manifestations [5]. Clinical reports have identified a spectrum of oral manifestations associated with COVID-19, including ulcerations without specific diagnosis, traumatic ulcers, erythema, petechiae, maculopapular rashes and vesiculobullous lesions [5]. In addition to non-specific manifestations, apthous-like and lichen-like lesions have been reported [5]. In COVID-19, TNF-α-mediated inflammation can cause mucosal damage that leads to apthous-like lesions. Instead, since the prevalence of lichen-like lesions in patients with COVID-19 infections is similar to that of lichen planus in the general population, it is still difficult to correlate the two pathologies [5].

Additionally, opportunistic infections, such as oral candidiasis, and herpetic reactivations have been frequently observed in COVID-19 patients [6]. In particular, oral candidiasis in these patients may arise due to prolonged use of broad-spectrum antibiotics, corticosteroids, immune suppression or the direct impact of SARS-CoV-2 on the oral mucosa [6]. Altered mucosal immunity related to the expression of ACE2 receptors in oral epithelial cells can facilitate fungal overgrowth and persistence [6].

Among oral manifestations related to COVID-19 disease, taste disturbances have emerged as prevalent symptoms, being the first recognized oral symptom of COVID-19 [7]. Taste dysfunctions in COVID-19 patients encompass a variety of clinical presentations, including hypogeusia (reduced taste), ageusia (complete loss of taste) and parageusia (distorted taste perception) [8]. These symptoms are often transient; however, in some cases, taste disturbances persist for weeks to months, affecting patients’ quality of life and nutritional status. In fact, one of the most persistent and troubling symptoms reported by COVID-19 survivors is long-term dysgeusia [9].

The connection between COVID-19 and oral health could be explained according to different hypotheses. As stated above, SARS-CoV-2 can directly affect the oral cavity due to the high expression of ACE2 receptors in the oral epithelium and salivary glands [3]. Moreover, the inflammatory response triggered by COVID-19, including a cytokine storm and endothelial dysfunction, can contribute to tissue damage and can facilitate the onset of immune-mediated oral lesions [3,4]. Therefore, oral manifestations may arise directly due to viral cytopathic effects, indirectly because of immune dysfunction or as sequelae of systemic conditions, comorbidities or adverse effects of therapeutic interventions [4].

As stated above, the oral manifestations of COVID-19 are very heterogeneous and, to date, there is no review that investigates all oral alterations related to COVID-19.

To best our knowledge, this study is the first that aims to provide a comprehensive overview of SARS-CoV-2-related oral manifestations, ranging from oral lesions to taste disturbances and osteonecrosis of the jaws, exploring the current knowledge regarding their incidence and clinical relevance in the context of COVID-19 infection.

## 2. Materials and Methods

### 2.1. Protocol, Registration and Search Strategy

A systematic review was conducted up to 2 September 2024, according to the Preferred Reporting Items for Systematic Reviews and Meta-Analyses (PRISMA) statement guidelines [10,11], using the databases PubMed and Scopus.

This review was registered at the International Prospective Register of Systematic Reviews (PROSPERO) database (CRD42025611551) on the 22nd January 2025. The search strategy is reported in Table 1.

### 2.2. Study Design

The research question as well as the eligibility criteria were defined following the acronym PEOS (Population, Exposition, Outcomes and Study design), being: (P) pediatric and adult patients; (E) SARS-CoV-2 infection with a positive laboratory test; (O) frequency of taste disturbances, frequency of oral manifestations, type and severity of oral lesions and frequency of osteonecrosis of the jaws; and (S) case-report, case-series, cross-sectional and observational studies.

### 2.3. Inclusion and Exclusion Criteria

Inclusion and exclusion criteria are reported in Table 2.

### 2.4. Study Selection

**Figure 1:**
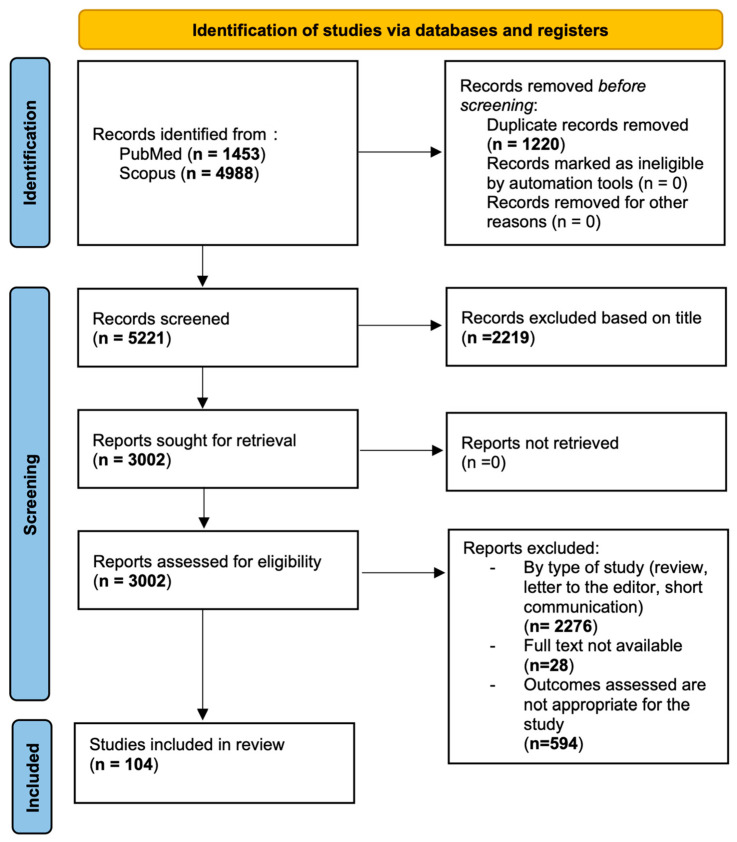
PRISMA flow-chart.

A flow-chart following PRISMA guidelines is presented in Figure 1. Only English language articles were identified which were published from March 2020 to 2 September 2024.

The article selection was completed by two authors in 2 steps. In step 1, two authors (AA and NC) separately screened the titles and abstracts of all the references through Rayyan free web application. Only those articles which matched the inclusion criteria were tabbed and the remaining publications were rejected. The concluding decision was taken in consultation with the third author (RG).

In step 2, we followed the same selection criteria and only those published full-text articles were selected which described the prevalence of all oral manifestations such as dysgeusia, oral mucosal lesions and osteonecrosis of the jaws in COVID-19 patients.

The same 2 authors (AA and NC) were associated independently in step 2. All selected articles were critically and intensely analyzed by 3 authors (AA, NC and RG) and the new publications were also chosen for selection analysis.

If there was any difference of opinion in either of the steps, it was settled by collective consensus among the 3 authors. Finally, only full-text articles were preferred and selected for this systematic review.

### 2.5. Data Extraction

At the outset, the first (AA) and second (NC) authors extracted the data from the chosen references. An extraction form was developed to list the essential information on the authors, name of the country, year of study, study design, number of subjects, mean age, gender, date of data collection, severity of COVID-19, period of appearance of oral manifestations, diagnostic methods used to evaluate clinical and radiographic symptoms and signs, results and conclusions of each study.

This was followed by the third author (RG) verifying the compiled data and affirmed their accuracy/preciseness/authenticity. If there was disagreement on any issue, it was resolved by discussion and mutual agreement among all the authors. In some articles, if the required information was missing, efforts were made to contact the authors of these publications, and the necessary data were filled in on an Excel sheet.

### 2.6. Quality Assessment and Critical Appraisal for the Systematic Review of Included Studies

The risk of bias of included studies was assessed by 2 authors (AA and NC) independently using a quality assessment checklist for prevalence studies, case reports, case series, case-control studies, and cohort and cross-sectional studies adapted by the Joanna Briggs Institute’s Critical Appraisal checklist [12,13]. In case of difference of opinion, the third author (RG) was consulted.

For each article, scoring was concluded only after consultation with all authors, and a study was specified as a high risk of bias when the ‘yes’ score was up to 49%, moderate when 50–69% and low when >70% [12,13].

## 3. Results

### 3.1. Study Characteristics

Initially, 6441 records were recognized from databases. After removing duplicate publications, 5221 references were left for title and abstract screening. After analyzing all the records, 3002 full-text articles were shortlisted for the second phase. Both the authors (AA and NC) completed full-text reading and excluded 2898 articles according to the eligibility criteria. The main reasons for excluding studies were study design (literature review/systematic review), oral manifestations related to vaccination and lockdown-related oral impairments. Finally, 104 studies [5,9,14,15,16,17,18,19,20,21,22,23,24,25,26,27,28,29,30,31,32,33,34,35,36,37,38,39,40,41,42,43,44,45,46,47,48,49,50,51,52,53,54,55,56,57,58,59,60,61,62,63,64,65,66,67,68,69,70,71,72,73,74,75,76,77,78,79,80,81,82,83,84,85,86,87,88,89,90,91,92,93,94,95,96,97,98,99,100,101,102,103,104,105,106,107,108,109,110,111,112,113,114,115,116] were selected for synthesis, of which 23 were case-report studies, 15 case series, 8 case-control and retrospective studies, 18 cohort studies and 40 cross-sectional and observational studies on dysgeusia, oral mucosal lesions and osteonecrosis of the jaws in COVID-19 patients.

### 3.2. Risk of Bias Within Studies

All included studies were assessed for risk of bias following Joana Briggs Institute guidelines and the observations are summarized in Supplementary Tables S1–S5.

Case reports, case series and case-control, cohort, retrospective, observational and cross-sectional studies were evaluated with the specified checklist for each study design [12,13].

Most prevalence studies (*n* = 46–47.8%) presented low risk of bias overall; however, 35 studies (35.3%) had moderate risk of bias. Only 23 studies (23.9%) had high risk of bias.

### 3.3. Summary of Clinical Findings

The main summary of the clinical findings of the included studies were divided according to the outcomes and reported in Table 3, Table 4 and Table 5, respectively. Some studies are reported twice because they included two or three different outcomes.

#### 3.3.1. Dysgeusia and Taste Alteration

The main findings of the included studies for dysgeusia and taste alteration [5,9,14,15,16,17,18,19,20,21,22,23,24,25,26,27,28,29,30,31,32,33,34,35,36,37,38,39,40,41,42,43,44,45,46,47,48,49,50,51,52,53,54,55,56,57,58,59,60,61,62,63,64,65,66,67,68,69] are reported in Table 3. COVID-19 patients showed a significantly higher prevalence of xerostomia and dysgeusia compared to non-infected individuals. The most common oral symptoms were xerostomia (45–74% of COVID-19 patients) and dysgeusia (32–59%), followed by loss of sweet (29.3%) and salt sensations (25.9%). Other symptoms included burning sensations (20.8%) and ageusia (19–66%). These symptoms were especially prevalent during the acute phase of the illness, with severe cases more likely to be associated with a positive COVID-19 test. Symptoms were more pronounced in younger individuals (over 15 years old) and those with a lower BMI (body mass index) and were influenced by sex and nationality. Anosmia and dysgeusia tended to linger the longest (up to 14.8%), with around 60% of patients improving within 3 months. In conclusion, dysgeusia and anosmia were prominent symptoms of COVID-19, with lasting effects, particularly among younger individuals and those with low BMI.

#### 3.3.2. Oral Mucosal Lesions

The main findings of the included studies for oral mucosal lesions [5,14,16,18,19,21,22,23,26,27,28,30,32,35,37,43,46,48,49,50,51,54,68,70,71,72,73,74,75,76,77,78,79,80,81,82,83,84,85,86,87,88,89,90,91,92,93,94,95,96,97,98,99] are reported in Table 4. Oral lesions were common, with ulcers being the most frequently reported (16.5% to 42.7% of patients). These were most found on the lips, tongue and buccal mucosa. Other oral manifestations included candidiasis (68% prevalence), herpes simplex, aphthous-like ulcers, geographic tongue, angular cheilitis, erythema and ulcerative necrotic lesions. Additional conditions included fissured tongue, exfoliative cheilitis, blisters, keratosis, white patches and petechiae. Oral ulcers were often hemorrhagic and necrotic, appearing as small ulcers, blood-filled bullae and erosive lesions, typically located on the tongue, lips, palate and buccal mucosa. Candidiasis frequently presented as white patches or fissures on the tongue and geographic tongue and crusted lips were also observed.

Oral ulceration was more common in male patients (19.4%) than females (14.8%), while tongue redness was more frequent in females (7.4%) than males (3.2%). Oral manifestations were more common in middle-aged patients, with 20.2% of children showing oral lesions, particularly mucositis (12.4%). Oral findings were strongly correlated with disease severity, with severe lesions (e.g., necrotic ulcers and candidiasis) more frequent in hospitalized patients, particularly those with COVID-19. Hemorrhagic ulcers and angular cheilitis were significantly associated with hospitalization (*p* < 0.05). Recurrent aphthous stomatitis was noted in 1.8% of patients, and other viral infections like herpes gingivostomatitis were also observed.

In conclusion, oral lesions, especially ulcers, candidiasis and herpes simplex infections, were common in COVID-19 patients. The prevalence of oral ulceration was particularly high on the lips, tongue and buccal mucosa, with more severe lesions seen in patients with advanced disease, particularly those hospitalized.

#### 3.3.3. Osteonecrosis of the Jaws

The main findings of the included studies on osteonecrosis of the jaws [100,101,102,103,104,105,106,107,108,109,110,111,112,113] are summarized in Table 5. A significant number of patients (76 individuals) with COVID-19-associated rhinomaxillary mucormycosis developed maxillary osteonecrosis (bone death) due to fungal infection. However, mucormycosis is not the sole cause of osteonecrosis. Other etiologies, including medication-related osteonecrosis of the jaw (MRONJ), osteoradionecrosis and trauma-related cases, should also be considered in discussions of maxillary bone necrosis.

Among the cases of mucormycosis-related osteonecrosis, diabetes mellitus was present in 93.4% of patients, and nearly all had received corticosteroid treatment during their COVID-19 management. While these factors are known to contribute to immunosuppression and increased susceptibility to invasive fungal infections, the reported percentage of affected individuals requires further context. A comparison with a control group of COVID-19 patients without osteonecrosis would help clarify the extent to which diabetes and corticosteroid use specifically increased the risk of mucormycosis-related osteonecrosis.

Osteonecrosis typically affected the maxilla and surrounding structures, including the zygomatic-maxillary process, hard palate and, in some cases, the mandible. Severe cases involved exposed necrotic bone, leading to complications such as tooth mobility and halitosis. Mucormycosis-related necrosis was often accompanied by necro-inflammatory changes, including the formation of an oroantral fistula with purulent exudate. In approximately 50% of patients, the disease extended to a rhino-orbital form, affecting both the maxilla and adjacent soft tissues.

Oral manifestations commonly included palatal discoloration, erosion and exposure of necrotic bone. Tooth mobility and halitosis were frequently observed due to maxillary bone involvement. Additionally, maxillary sinus involvement was documented in 30% of cases unilaterally and 15% bilaterally, further complicating the clinical presentation.

In conclusion, maxillary osteonecrosis in the context of COVID-19-related mucormycosis represents a severe complication, particularly in patients with underlying diabetes mellitus and those who received corticosteroids. However, osteonecrosis can arise from multiple etiologies, and future studies should aim to differentiate mucormycosis-induced cases from other forms. Early detection and prompt management of mucormycosis, alongside addressing predisposing conditions, remain crucial in mitigating these complications.

## 4. Discussion

The aim of this study was to provide a comprehensive overview of SARS-CoV-2-related oral manifestations, including taste disturbances, oral lesions and osteonecrosis of the jaws. While some other reviews have addressed SARS-CoV-2-related oral manifestations [114,115,116,117], they have not offered a holistic perspective on the diverse impacts on oral health, such as neurological effects, oral lesions and bone involvement.

Taste disturbances, as dysgeusia, have emerged as prevalent symptoms being the first recognized oral symptom of COVID-19. The mechanism behind COVID-19-induced dysgeusia is not fully understood, but several potential pathways have been proposed based on current research [118,119]. SARS-CoV-2 primarily enters human cells through the ACE2 receptor, which is expressed on various cell types, including those in the oral cavity and tongue [3]. Recent studies suggest that the virus may infect cells within the taste buds, particularly those expressing ACE2 receptors, leading to damage or disruption of taste function [120,121]. The presence of ACE2 receptors in the epithelial cells of the tongue and oral cavity suggests that viral particles might directly interfere with taste receptor cells, altering taste perception [122]. Besides the local effect, SARS-CoV-2 has been shown to affect the central nervous system, including areas involved in taste perception [123]. The virus may enter the brain through the olfactory nerve or via blood circulation, causing disruptions in neural pathways related to taste [124]. Some studies have suggested that damage to the brain regions involved in taste processing, such as the gustatory cortex, could contribute to dysgeusia [125,126]. While both local and central mechanisms are likely involved, their relative importance remains debated. Direct viral damage to taste bud cells may play a more immediate role in early dysgeusia, whereas central nervous system involvement could contribute to prolonged or persistent taste alterations. Further research is needed to clarify the dominant mechanisms and their clinical significance [125].

About oral manifestations, oral ulcers were the most frequently reported lesion (16.5% to 42.7% of patients). Possible mechanisms behind COVID-19-related oral lesions are direct viral infection, immune system dysregulation, medication-induced lesions or stress and psychological factors. In detail, SARS-CoV-2 may directly infect cells in the oral cavity, including those in the tongue, salivary glands and mucosal tissues, through the ACE2 receptors. The virus could trigger local inflammation, leading to the development of oral lesions like ulcers or vesicles [127,128].

The immune response to COVID-19, particularly the cytokines, may play a significant role in the development of oral lesions. Excessive inflammation can disrupt oral mucosal integrity, leading to the formation of lesions such as aphthous ulcers or erythema multiforme [129]. Some of the medications used to treat COVID-19, such as corticosteroids, antivirals and antibiotics, can cause oral mucosal changes. Corticosteroids, for example, can weaken the immune system, predisposing individuals to secondary infections like candidiasis. Other medications might directly irritate the oral tissues, leading to ulceration or other forms of oral mucositis [130]. The psychological burden of COVID-19, including stress, anxiety and isolation, can exacerbate conditions like aphthous stomatitis or other inflammatory oral lesions. Stress is known to affect immune function and may trigger flare-ups of pre-existing conditions like herpes simplex or lichen planus [131]. Moreover, COVID-19 infection may affect the salivary glands, leading to dry mouth (xerostomia). Reduced salivation can compromise the mucosal barrier, making the oral tissues more susceptible to infections and irritation. This may facilitate the development of candidiasis or increase the risk of ulcers and other lesions [132].

Osteonecrosis of the jaws has emerged as a significant complication in the context of COVID-19, garnering increased attention due to its severe consequences on bone health. While mucormycosis, or “black fungus”, has been prominently associated with ONJ, other factors also contribute to the development of this condition, and these should be addressed comprehensively. Osteonecrosis of the jaws is a condition characterized by the death of bone tissue due to insufficient blood supply, leading to tissue damage and potential infection. One of the key contributing factors in the context of COVID-19 is the prolonged use of corticosteroids. These medications can impair bone remodeling by reducing osteoblast activity and enhancing osteoclast activity. As a result, the bone structure becomes weakened, increasing the susceptibility to osteonecrosis, particularly in patients who have received long-term steroid therapy as part of their COVID-19 treatment regimen [133].

Additionally, COVID-19-induced immune dysregulation further predisposes patients to infections, which can compromise bone health and exacerbate the development of osteonecrosis. The compromised immune system can hinder the body’s ability to fight infections, allowing bacterial or fungal agents to invade bone tissue, causing further damage. Secondary infections, such as those caused by bacteria or fungi, play a critical role in worsening bone damage, especially in individuals with pre-existing bone vulnerability due to corticosteroid use or other factors [134].

Mucormycosis, caused by fungi from the Mucorales order, is one of the most significant and aggressive infections associated with ONJ. It typically affects immunocompromised individuals, and its occurrence has been increasingly reported in COVID-19 patients. The condition is characterized by rapid tissue necrosis, and the fungus can invade jawbone tissue, leading to extensive damage. The risk of developing mucormycosis is elevated by factors such as corticosteroid use, which weakens immune defense, as well as underlying hypoxia, acidosis and prolonged oxygen therapy. The humid environments of hospitals and exposure to hospital-acquired infections also heighten the likelihood of fungal invasions, particularly in patients who are hospitalized for extended periods [135].

However, mucormycosis is not the only cause of ONJ in the COVID-19 context. In addition to corticosteroid therapy and immune dysregulation, other factors such as pre-existing dental conditions, poor oral hygiene and comorbidities (e.g., diabetes, hypertension) can contribute to the onset of osteonecrosis. Additionally, medications other than corticosteroids, such as antivirals and immunosuppressive drugs used to manage COVID-19, may also play a role in increasing the risk of ONJ by further impairing immune function or promoting bone fragility. A more comprehensive understanding of these factors, including the roles of various medications, will be crucial in mitigating the risk of osteonecrosis in COVID-19 patients and optimizing patient care.

Some relevant clinical implications may be drawn by these findings: healthcare professionals should be vigilant for oral manifestations in COVID-19 patients, as these symptoms can aid in early diagnosis and management; regular oral assessments are crucial for COVID-19 patients; effective management of these oral manifestations may require collaboration between medical and dental professionals to ensure comprehensive care.

Although this study provides a comprehensive evaluation of COVID-19-related oral manifestations, several limitations need to be addressed. First, while there is a growing body of evidence suggesting that COVID-19 may contribute to certain oral manifestations, establishing a definitive cause-effect link is challenging due to the involvement of multiple factors. More focused research is needed to better understand the direct impact of SARS-CoV-2 on oral health and to differentiate it from other contributing factors such as medication use, stress and changes in oral hygiene practices. Second, the heterogeneity of the studies with varying designs, methodologies, sample sizes and populations do not allow a meaningful synthesis of the data. In this case, studies differed in terms of how oral symptoms are defined, diagnosed, or measured. Moreover, 58 out of 104 studies had moderate or high risk of bias. The risk of bias in the included studies can significantly influence the interpretation of the results, as it can affect the reliability and generalizability of the findings. In our study most prevalence studies (*n* = 46–47.8%) presented low risk of bias overall, and 35 studies (35.3%) had moderate risk of bias. Only 23 studies (23.9%) had high risk of bias. Since a significant portion of studies had low or moderate risk of bias, the overall findings may still be reliable, offering a stronger foundation for any conclusions drawn.

## 5. Conclusions

COVID-19 has been associated with a range of oral manifestations, including dysgeusia, various oral lesions and osteonecrosis of the jaw. The complex interplay of viral infection, immune response, medication use and stress likely contributes to these oral complications. Early recognition and management of these oral manifestations are crucial for improving patient outcomes, and further research is needed to fully understand their pathophysiology and to develop targeted preventive and therapeutic strategies for COVID-19-related oral health issues. Moreover, preventive measures such as routine oral assessments in COVID-19 patients, especially those hospitalized or receiving long-term medications, could significantly reduce the risk of severe oral complications.

## Figures and Tables

**Table 1:** Search strategy.

Database	Search Strategy	Hits
PubMed	(“long COVID*”[All Fields] OR “long-term COVID*” [All Fields] OR “COVID*”[All Fields] OR “Coronavirus Disease*” [All Fields]) AND (“oral health” [Title/Abstract] OR “oral disease” [Title/Abstract] OR “oral manifestation*” [Title/Abstract] OR “oral lesion*” [Title/Abstract] OR “osteonecrosis” [Title/Abstract] OR “dysgeusia” [Title/Abstract])	1453
Scopus	(TITLE-ABS (long COVID*) OR TITLE-ABS (long-term COVID*) OR TITLE-ABS (COVID*) OR TITLE-ABS (“Coronavirus Disease*”) AND (TITLE-ABS (oral health) OR TITLE-ABS (oral disease) OR TITLE-ABS (oral manifestation*) OR TITLE-ABS (oral lesion*) OR TITLE-ABS (osteonecrosis) OR TITLE-ABS (dysgeusia))	4988

**Table 2:** Inclusion and exclusion criteria.

**Inclusion Criteria**
All the observational, case-series, case-report, cross-sectional studies and studies published in an international peer-reviewed journal on oral manifestations related to COVID-19 disease and long-COVID disease
Only English language articles published from March 2020 to September 2024
Oral manifestations included taste disorders (dysgeusia, ageusia and hypogeusia), xerostomia, oral mucosal lesions like aphthous ulcers, geographic tongue, candidiasis, erythema multiforme-like lesions, herpes zoster, herpes simplex, oral lichen planus, lichenoid lesions and osteonecrosis of the jaws with or without opportunistic infections like mucormycosis and aspergillosis.
**Exclusion criteria**
All systematic reviews, narrative reviews, editorial letters, communications, posters or abstracts
Articles not written in English
Studies based on oral manifestation after COVID-19-vaccination and oral impairment due to lockdown

**Table 3:** **Study Characteristics (*taste disorders*):** M, male; F, female; ENT, ear, nose and throat; OGD, oesophago-gastro-duoden; NHANES, National Health and Nutrition Examination Survey; rRT-PCR, reverse transcription polymerase-chain-reaction; CSF, cerebro-spinal fluid; PCR, polymerase-chain-reaction; OR, odds ratio; CI, confidence interval, ICD, International Classification of Diseases; NAATs, nucleic acid amplification tests; VAS, visual analogic scale; SNOT-22, Sino-Nasal Outcome Test-22; MRI, magnetic resonance imaging.

First Author, Year, Reference and Country	Type of Study	Total. *n*. of Patients, Age, Gender	Date Data Collected	COVID-19 Severity and Latency (Days)	Period of Appearance	Diagnostic Method	Results	Conclusions
Al-Magsoosi M.J.N. et al., 2023 [14] Basrah, Iraq	Cross-sectional	574 patients aged 18–79 (M = 196, F = 378)	From October 2021 to April 2022	Mild–moderate	1–6 months after diagnosis of COVID-19	Questionnaire to collect information from each patient.	Dry mouth (83%), gustatory changes (46%), burning sensation (20.8%), ageusia (66.8%).	There is a positive correlation between the incidence of oral signs and symptoms associated with COVID-19 infection and the severity of the infection.
Ali F.A. et al., 2023 [9] Enniskillen, Northern Ireland, UK	Cross-sectional	405 patients, aged 38.2 ± 11.3 (M = 220, F = 185)	From October 2020 to June 2021	Mild–moderate	Not reported	Anosmia Reporting Tool and brief version of the questionnaire of olfactory disorders.	Sex and nationality of participants were significantly associated with anosmia and dysgeusia (*p* < 0.001).	Anosmia and dysgeusia are prevalent symptoms of COVID-19 disease, especially among females.
AlShakhs A. et al., 2022 [15] AlAhsa, Saudi Arabia	Observational	274 patients, aged 18–65 (M = 124, F = 150)	From 5 June to 30 July 2020	Mild (33.9%), moderate (54.7%), severe (11.3%)	1 week to 2 months	Questionnaire of Olfactory Disorders–Negative Statements (sQODNS)	The most common ENT-related symptoms were headache 69%, insomnia 65.3% and dysgeusia 64.6%.	The duration of both insomnia and dysgeusia is an important contributing factor on the patient’s psychological state as it may prolong their isolation period.
Binmadi N.O. et al., 2022 [16] Jeddah, Saudi Arabia	Cross-sectional	195 patients, aged 18–70 (M = 48, F = 147)	From October 2021 to March 2022	19% mild, 61% moderate 19% severe.	1–7 days after the onset of COVID-19 symptoms	Patient interview and clinical history collection	A total of 57 (29%) had oral manifestations; the most common were taste disorders (60%) and xerostomia (42%).	Oral manifestations of COVID-19 were common among female patients and linked to certain general COVID-19 symptoms regarding frequency and extent.
Borah H. et al., 2022 [17] Assam, India	Prospective	2000 patients, aged 0–80 (M = 1150, F = 850)	Not reported	Not reported	Not reported	Clinical examination and clinical history collection	Hyposmia/anosmia (880 cases= 44%) and dysgeusia (640 cases= 32%)	ENT manifestations should be kept in mind while making the diagnosis of COVID-19.
Chawla J. et al., 2022 [18] Mangalagiri, India	Cross-sectional	367 patients, aged 18–60 (M = 257, F = 110)	From September to December 2020	Mild–moderate	Not reported	Patient interview, clinical history collection and intra and extra oral objective examination.	Xerostomia and dysgeusia were significantly higher in COVID-19 positive patients.	Xerostomia and dysgeusia are the common oral manifestations of COVID-19.
Dalipi S.Z. et al., 2021 [19] Prishtina, Kosovo	Case report	17-year-old male patient	Not reported	Moderate-severe	2 weeks after diagnosis	Clinical history collection and intra and extra oral objective examination.	Xerostomia, loss of taste and dysgeusia.	The diagnosis of this case-report is of exudative erythema multiforme.
Eita A.A.B. et al., 2021 [20] Alexandria, Egypt	Case report	31-year-old female patient	From April to May 2021	Moderate	2–4 weeks after diagnosis of COVID-19.	Clinical history collection and intra and extra oral objective examination.	Dysgeusia is the main symptom.	Post-acute COVID-19 syndrome can show oral manifestations.
El Kady D.M. et al., 2021 [21] Cairo, Egypt	Cross-sectional	58 patients, aged 18–46 (M = 31, F = 27)	From 15 May to 10 June 2020	Not reported	Not reported	Online questionnaire created by Google Form.	The highest prevalence symptoms are dry mouth 39.7% (*n* = 23), gustatory dysfunction as 34.5% (*n* = 20) loss of salt sensation, 29.3% (*n* = 17) loss of sweet sensation and 25.9% (*n* = 15) altered food taste.	COVID-19 significantly impacts the oral cavity and salivary glands, as salivary gland-related symptoms and taste disorders are highly prevalent in COVID-19 patients.
El Tantawi M. et al., 2022 [22] Alexandria, Egypt	Cross-sectional	5342 patients, aged 18–23 (M = 2219, F = 3123)	From August 2020 to January 2021	Not reported	Not reported	Questionnaire completed by the patients.	42.7% of participants reported oral manifestations. Dry mouth (11.1% vs. 7.5%, *p* = 0.009) and change in taste (11.5% vs. 2.7%, *p* < 0.001) were greater in COVID-19 infected than non-infected persons.	Dry mouth and changed taste may be used as an indicator for COVID-19 infection in low COVID-19-testing environments.
Emelyanova N. et al., 2021 [23] Kharkiv, Ukraine	Case-report	38-year-old female patient	September 2020	Not reported	Not reported	Clinical history collection and intra and extra oral objective examination.	Dryness in the oral cavity and burning tongue were the main symptoms.	Importance of a dentist as part of a multidisciplinary team in diagnostics and treatment of COVID-19 was noted.
Falaki M. et al., 2022 [24] Tehran, Iran	Case-control	189 patients divided into case and control groups based on the presence of olfactory and gustatory symptoms (aged 21–96; M = 107, F = 82).	From January 2021 to March 2021	Moderate-severe	Not reported	Collection of clinical and medical history data.	Taste disorders were found in 24.3% of patients, ageusia in 19% and dysgeusia in 5.3%.	OGD symptoms can be used to detect COVID-19-infected patients.
Fantozzi P.J. et al., 2020 [25] Rome, Italy	Cohort	111 patients (F = 53, M = 58), a median age of 57 (range: 48–67).	From 6 March to 30 April 2020	Not reported	3–7 days after COVID-19 diagnosis.	A modified survey obtained from the NHANES 2013–2014 for taste and smell disorders and the Fox Questionnaire for dry mouth.	Xerostomia (45.9%), dysgeusia (59.5%)	The study showed that xerostomia, olfactory and gustatory dysfunctions are common symptoms reported as concomitant, and in some cases the sole manifestation of COVID-19.
Favia G. et al., 2021 [26] Bari, Italy	Observational	123 patients (F = 53, M = 70), median age of 72 years.	From October to December 2020	Moderate (77%), severe (17%) and critical (6%).	Not reported	Intraoral examination	Dysgeusia (64%) Hypogeusia (27%) Ageusia (9%)	This study on a large series highlighted that oral lesion in more than half of cases (65.9%) occurred in the early stage of COVID-19 before the beginning of specific therapies.
Ferdeghini C. et al., 2022 [27] Monza, Italy	Observational	58 patients aged 22–92 (F = 20, M = 38)	From April 2021 to May 2021	Severe	0–10 day after COVID-19diagnosis	Intraoral examination	Ageusia (27.6%) and the metallic taste of water and food (8.6%).	This study therefore seems to demonstrate a possible correlation between the alterations found in patients hospitalized for COVID-19 and the infection itself by the virus.
Fernandes T.J. et al., 2023 [28] Rio de Janeiro, Brazil	Cross-sectional	136 patients, aged 37 ± 14.13 (M = 57, F = 79) divided into 2 groups: rRT-PCR-positive and rRT-PCR-negative patients.	From October 2020 to September 2021	Not reported	Not reported	Intra and extraoral objective examination and intervist by dentists.	Xerostomia (*n* = 85; 62.5%) dysgeusia/ageusia (*n* = 57; 41.9%), halitosis (*n* = 7; 13.0% vs. *n* = 1; 1.2%; *p* = 0.007).	A high prevalence of oral manifestations was observed in symptomatic patients with suspected or confirmed COVID-19.
Flores-Silva F.D. et al., 2021 [29] Mexico City, Mexico	Observational	1072 patients, aged 53.2 ± 13 (M = 697, F = 375).	From 15 March to 30 June 2020	Moderate-severe	Not reported	Clinical history collection	Dysgeusia (8%) and anosmia (7%) were the most common neurologic symptoms at hospital presentation.	The high frequency of neurologic manifestations during hospitalization in COVID-19 patients suggested a potentially high burden of short and long-term sequelae these for these patients.
Ganesan A. et al., 2022 [30] Rajasthan, India	Cross-sectional	500 patients, aged 53.46 ± 17.50 (M = 367, F = 133)	From 18 October to 7 November 2020	Mild	Not reported	Intra and extra oral objective examination.	Almost 51.2% of patients presented dry mouth with gustatory disturbance, 28% with xerostomia.	COVID-19 is found to effect oral health with greater probability in patients with severe diseases.
Gençeli M. et al., 2022 [31] Konya, Turkey	Retrospective	467 patients, aged 1 month to 18 years (M = 242, F = 224)	From March 2020 to April 2021	Mild–moderate	Not reported	Demographic data, epidemiologic history, symptoms, contact history, laboratory tests and treatments were recorded.	Dysgeusia (11%), anosmia (14.6%) in those aged over 15 years were found to be significantly more common in comparison with the other age groups (*p* < 0.05).	While SARS-CoV-2 infection may be asymptomatic and COVID-19 usually has a mild clinical course, some children have severe disease or mortality.
Glavina A. et al., 2024 [5] Split, Croatia	Case series	15 patients, aged 24–85 (M = 6, F = 9)	From November 2020 to January 2024	Mild-severe	Not reported	Intra and extra oral objective examination.	Erosion, erythema and atrophy at tongue (tip, dorsum and anterior half), hard palate and buccal mucosa occurred in middle-aged patients, with an equal distribution by sex.	Oral lesions presented in a mild form and did not correlate with the severity of the clinical picture of COVID-19.
Gogotishvili M. et al., 2024 [32] Batumi, Georgia	Cohort	55 patients, aged 18 to 89 (M = 30, F = 25)	Not reported (during the COVID-19 pandemic)	Mild–moderate	Not reported	Intra and extra oral objective examination	Pain and burning on mouth (36.4%), alteration or total loss of taste (60%), xerostomia (27.3%), dysgeusia and oral burning (68.2% and 77.3%).	Oral mucosal alterations and lesions were prevalent in this series of COVID-19 patients.
Hajare Priti S. et al., 2022 [33] Karnataka, India	Cross-sectional	167 patients, aged 17–70 (M/F = 2:1)	From July to September 2020	Not reported	14–30 days after diagnosis of COVID-19.	Standardized questionnaire of 22 questions regarding olfactory and gustatory dysfunction.	98 patients (58.58%) had altered sense of taste and dysgeusia.	Smell and taste loss has a high prevalence in patients of COVID-19 and health care workers should keep high degree of suspicion for COVID-19 when patients present with these symptoms.
Haran J.P. et al., 2021 [34] Massachusetts, USA	Prospective	27 patients, aged 62 ± 14.3 (M = 19, F = 8)	From April 2020 to February 2021	Moderate-severe	8–12 weeks after infection	Clinical history collection	The symptoms that lasted the longest were ageusia and anosmia (14.8%).	Our findings suggest an association with the oral microbiome and long-COVID, revealing the possibility that dysfunction of the oral microbiome may have contributed to this draining disease.
Hussain S. et al., 2023 [35] Peshawar, Pakistan	Cross-sectional	117 patients, aged 15–70 (M = 85, F = 32)	From 1 October 2020, to 30 January 2021	Not reported	10.62 ± 6.19 days after infection.	Clinical history collection and intra and extra oral objective examination.	Taste alterations were experienced by 49.6% (*n* = 58) xerostomia 34.2% (*n* = 40).	Oral manifestations were observed in 56% of PCR- confirmed COVID-19-positive patients, with taste alterations being the most common at 49.6%.
Jain A. et al., 2020 [36] Faridabad, India	Cross-sectional	410 patients (F = 36.1%, M = 63.9%); a mean age of 37.21 ± 13.30 years	From May 2020 to June 2020	Mild to moderate	Not reported	Questionnaire to collect information from each patient.	Dysgeusia (22.4%)	Olfactory and taste dysfunction are significant symptoms in the clinical presentation of coronavirus disease–19.
Johansson A.K. et al., 2023 [37] Bergen, Norway	Cohort	8203 patients, aged 89–90 (M = 3662, F = 4541)	April 2022	Mild–moderate	16 months (range 5–28 months)	Oslo COVID-19 questionnaire	Dry mouth, taste change and burning on buccal mucosa.	Long-COVID general and orofacial symptoms are common among older elderly COVID-19 survivors.
Kajita M. et al., 2021 [38] Takasaki, Japan	Case-report	50-year-old woman patient	Not reported	Moderate	6 days after testing positive for infection	Clinical history collection, intra and extra oral objective examination, neurological examination, laboratory and urine tests	Dysgeusia and olfactory abnormality.	Dissociation in CSF, neurological examinations and nerve conduction study findings, finally diagnosed the patient with Guillain-Barré syndrome
Klopfenstein T. et al., 2020 [39] Trevenans, France	Retrospective	70 patients, aged 57.0 ± 19 (M = 29, F = 41)	From 1 March to 14 March 2020	Mild–moderate	7–28 days after infection	Clinical history collection	37 (53%) patients had anosmia, which was associated with dysgeusia in 81% of cases.	Anosmia can be an unknown neurologic symptom in COVID-19. More than half of patients with COVID-19 have anosmia.
Köseoğlu Toksoy C. et al., 2021 [40] Afyonkarahisar, Turkey	Prospective	379 patients, aged 52 ± 17.5 (M = 187, F = 192)	From July to October 2020	Moderate-severe	Not reported	Clinical history examination and objective examination of the whole body.	The most common neurologic symptoms are myalgia (48.5%), headache (39.6%), anosmia (34.8%) and dysgeusia (34%).	The prevalence of neurological symptoms is very high in patients with COVID-19.
Kumar L. et al., 2021 [41] Faridabad, India	Prospective	141 patients (M = 58.9%, F = 41.1%), mean age of 15.2 years (range 10–19).	From May to August 2020	Not reported	Not reported	ENT consultation.	Dysgeusia (24.1%)	Loss of smell and taste are common symptoms in COVID-19 patients and may be the only symptoms in some patients. These symptoms may help in early diagnosis of COVID-19 patients and in reducing the spread of infection
Martinez F. et al., 2022 [42] Viña del Mar, Chile	Cross-sectional	2,187,962 patients, aged 43.1 ± 17.5 (M = 47.2%, F = 52.8%)	From March 2020 to January 2021	Not reported	Not reported	Collection patient’s data from national registry.	The most specific features of disease were anosmia and dysgeusia/ageusia.	No single clinical feature can fully confirm or exclude an infection by SARS-CoV-2 but the combination of both demographics can identifying patients with the disease.
Metin N. et al., 2024 [43] Erzurm, Turkey	Cross-sectional	383 patients, aged 39 ± 12 (M = 214, F = 168)	From 1 January to 30 June 2020	Not reported	1–3 months	Intra and extra oral objective examination and PCR test.	Xerostomia, loss of taste, dysgeusia, oral burning and dry mouth, were significantly higher in patients with positive PCR tests.	There is a significant correlation between COVID-19 infection, PCR positivity and the numerous manifestations of oral lesion.
Miyazato Y et al., 2022 [44] Tokyo, Japan	Cross-sectional	457 patients, aged 39–55 (M = 226, F = 231)	Between February 2021 and March 2021	Mild (84.4%), moderate (12.7%), severe (2.9%)	6–12 months after diagnosis	Questionnaire and clinical history collection	Younger age and low body mass index were factors for developing dysgeusia (OR: 0.98, 95%CI: 0.96–1.00 and OR: 0.93, 95%CI: 0.88–0.98, respectively).	Many patients, even those with mild conditions, experience long-term residual symptoms.
Mizrahi B. et al., 2023 [45] Tel Aviv, Israel	Cohort	1,913,234 patients, aged 5–60 years (M = 945,137, F = 968,096)	From 1 March 2020 to 1 October 2021	Mild	30–360 days after infection	Intra and extra oral objective examination using symptoms and diagnoses recorded by physicians as ICD-10.	Anosmia and dysgeusia have a hazard ratio in early period of 2.96 (2.29 to 3.82) and 11.0 (8.5 to 13.6) in late period.	COVID-19 infection was significantly associated with increased risks in early and late periods for anosmia and dysgeusia.
Mohammed F. et al., 2023 [46] Dammam, Saudi Arabia	Observational	179 patients, aged 10–51 (M = 102, F = 77)	From August to December 2020	Mild–moderate	1–7 weeks	Questionnaire by QuestionPro software.	Xerostomia (22.1%), burning sensation on lips, gingiva, retromolar area, anterior and posterior tongue (11.3%), xerostomia with burning sensation (8.9%).	The presence of oral mucosal lesions and dysgeusia or dry mouth and a burning sensation, along with COVID-19 generic symptoms, should be regarded as suggestive but not definite markers of COVID-19.
Munsch N. et al., 2022 [47] Vienna, Austria	Cohort	9133 patients, aged 31–40	From 2 November 2020 to 18 November 2021	Mild–moderate	Not reported	Patients answered 12 yes/no questions about symptoms to assess their risk for COVID-19.	Symptoms significantly associated with a positive COVID-19 test were dysgeusia (28.9%) and hyposmia (26%).	The study provides reliable COVID-19 symptom statistics based on the general population verified by NAATs.
Muthyam A.K. et al., 2022 [48] Telangana, India	Observational	100 patients, aged 20–73 (M = 51, F = 49)	Not reported	Mild–moderate	2 weeks to 3 months after diagnosis of COVID-19.	Questionnaire: a close-ended, validated, questionnaire containing 25 questions.	Xerostomia is the commonest symptom (44%), followed by gum bleeding (6%) and burning sensation (4%).	Xerostomia, frequent aphthous ulcers, swallowing difficulty and burning mouth were the most frequently encountered symptoms in study subjects during the disease and post recovery.
Naser A.I. et al., 2021 [49] Mosul, Iraq	Prospective	338 patients (M = 200, F = 138)	From August 2020 to March 2021	Not reported	Not reported	Extra and intraoral examination and radiographic evaluation (if needed).	Loss of taste (79.5%) dryness of the oral cavity and burning (56.7%).	Most of the intraoral and extraoral lesions are temporary and disappear with appropriate treatment in COVID-19 patients, so early treatment is quite important to decrease complications.
Natto Z.S. et al., 2021 [50] Jeddah, Saudi Arabia	Cross-sectional	109 patients, aged 39.3 ± 12.4 (M = 76, F = 36)	From 28 July to 5 October 2020	Mild	2–15 days after infection	Questionnaire and clinical examination.	Loss of taste is the most common oral manifestation of COVID-19 (43.4%).	Oral examinations of COVID-19 patients should be conducted as part of routine examinations to investigate any possible correlation between the disease and the oral cavity.
Nikalje M.R. et al., 2021 [51] Maharashtra, India	Observational	201 patients (M = 150, F = 51)	From June 2020 to September 2020	Not reported	Not reported	Routine screening procedure with the help of questionnaires.	Anosmia (25.9%), ageusia (33.3%), decreased salivation (27.9%), burning sensation in the mouth (1%).	Chemosensitive changes like ageusia and anosmia should raise the suspicion of COVID-19 infection in the mind of the clinician.
Otsuka Y. et al., 2021 [52] Okayama, Japan	Cohort	87 patients, aged 26.5–53.0 (M = 41, F = 46)	From 15 February to 17 September in 2021	Moderate-severe	0–112 days after diagnosis of COVID-19	Patient interview and clinical history collection.	Dysgeusia is one of the most frequent symptoms and was referred in 23 patients (26.4%).	General physicians skilled in using a comprehensive approach would be optimal to see patients with such complex symptoms.
Paszynska E. et al., 2023 [53] Poznan, Poland	Cross-sectional	120 patients, aged 74.4 ± 15.4 (M = 50, F = 70)	From January to March 2022	Moderate-severe	1–4 weeks after infection	Intra and extra oral objective examination	Xerostomia (74.2%), oral atrophy and inflammation (80.8%).	COVID-19 hospitalized patients with severe symptoms crossing with poor oral health-related conditions.
Patel D. et al., 2024 [54] Portsmouth, UK	Cross-sectional	104 patients, aged 18–79 (M = 8, F = 93, other = 3)	From February to May 2022	Mild–moderate	From 1–7 days to more than 3 months.	Self-reported online questionnaire and healthcare professionals ’semi-structured interviews.	Burning feeling in mouth, tongue and lips (25%), xerostomia (37.5%), change in taste and/or smell (45%).	Healthcare professionals have observed oral manifestations in individuals diagnosed with long-COVID.
Ramasamy K. et al., 2020 [55] Negeri Sembilan, Malaysia	Observational	145 patients, aged 43.0 ± 17.7 (M = 90, F = 55)	From 23 March to 2 May 2020.	Mild–moderate	Until 2–3 weeks after infection.	Clinical history collection and completion of questionnaire.	31 patients (21.4%) reported olfactory dysfunction and 34 (23.4%) reported dysgeusia. 6 patients (13.6%) reported isolated sudden-onset anosmia.	Olfactory and gustatory dysfunction is a pertinent manifestation of COVID-19 and long-COVID-19.
Rogn A. et al., 2024 [56] Oslo, Norway	Cross-sectional	100 patients, aged 29–55	From October 2020 to June 2023	Mild–moderate	Not reported	Oslo COVID-19 questionnaire and VAS scale.	Parosmia (80%), hyposmia (42%), anosmia (53%), dysgeusia (34%), ageusia (3%). Burning sensation on anterior, lateral and whole tongue and on palate, throat, lips and buccal mucosa.	Post-COVID-19 patients experience a range of chemosensory, trigeminal and salivary disturbances, occurring in various combinations.
Sakurada Y. et al., 2022 [57] Okayama, Japan	Retrospective	65 patients, aged 25–56 (M = 29, F = 36)	From February to July 2021	Mild: 76.9%; Moderate: 12.3%; Severe: 4.6%	1–3 months after infection	Clinical history collection and intra and extra oral objective examination.	Dysosmia, dysgeusia, sleeplessness and headache improved in about 60% of the patients after 3 months.	Long-COVID symptoms were improved at 3 months after the initial visit in more than half of the patients.
Samaranayake L.P. et al., 2020 [58] Hong Kong, China	Cohort	149 patients, aged 42.6 ±15.1 (M = 101, F = 48)	From April to June 2020	Moderate-severe	Not reported	SNOT-22 questionnaire.	Mild cases: anosmia 87%; dysgeusia 4%; anosmia and dysgeusia 9%. Moderate cases: anosmia 72%; dysgeusia 22%; anosmia and dysgeusia 6%.	Data confirm the commonality of chemosensory dysfunction during COVID-19 progression.
Schambeck S.E. et al., 2021 [59] München, German	Cohort	44 patients, aged 23–62 (M = 15, F = 29)	From 1 April to 10 June 2020	Mild (54.5%) Moderate (29.5%) Hospitalized (2.3%)	21–112 days after infection	Questionnaire Q1 and Q2	11 (25.0%) participants experiencing dysgeusia, 5 mentioned that they lost the ability to differentiate tastes in mixed dishes.	COVID-19-induced phantosmia, parosmia and dysgeusia can persist for longer periods.
Sequeria Rodriguez P. et al., 2023 [60] Jalisco, Mexico	Case-report	25-year-old female patient	Not reported	Moderate	3,5 months after diagnosis of COVID-19	Intra and extra oral objective examination and cranial MRI.	Dysgeusia (32.4%), anosmia (17%) and parosmia (8%) are correlated to COVID-19 infection.	Dysgeusia has been reported that it reaches up to 32.4% as a problem or late symptom.
Sheng W.H. et al., 2021 [61] Taiwan	Observational	217 patients (F = 53.5%, M = 46.5%), median age 33 years (24–50)	From 22 January to 7 May 2020	Not reported	Not reported	Clinical history collection	Dysgeusia (28.6%)	Dysosmia and/or dysgeusia are pertinent clues for the diagnosis of COVID-19, particularly in the early stage of the disease.
Singer-Cornelius T. et al., 2021 [62] Lucerne, Switzerland	Cross-sectional	41 patients, aged 35.22 ± 13.5 (M = 10, F = 31)	From 3 March to 12 May 2020	Mild–moderate	Not reported	Questionnaire and objective taste and smell test.	3.1% suffered from total hypogeusia, 2.6% from ageusia. A significant loss of sour (33.3% (13/39)) and salty taste (17.9% (7/39)) could be recognized.	Most patients suffering from objective dysgeusia present a deficit in sour and salty taste.
Skrypnikova T. et al., 2022 [63] Poltava, Ukraine	Case series	29 patients, aged 46.5 ± 13 (M = 12; F = 17)	From February 2020 to December 2021	Mild–moderate (70%) Severe (30%)	2–8 months after infection.	Clinical history collection, intra and extra oral objective examination and laboratory tests.	Primary concerns and symptoms in the oral mucosa were xerostomia (17.2%) and burning mouth (13.8%).	After suffering COVID-19 on the background of chronic systemic diseases, it is expected long term incidence of oral mucosa morbidity for 8 months, with a significant prevalence of chronic candidal glossitis.
Sørensen A.I.V. et al., 2022 [64] Copenhagen, Denmark.	Cross-sectional	152,880 patients, aged 49 ± 34 (M = 59,341, F = 93,494)	From September 2020 to April 2021	Not reported	Around test date until 6–12 months after test.	Nationwide questionnaire.	The largest adjusted risk differences (RD) were observed for dysosmia (RD = 10.92%, 95% CI 10.68–11.21%), dysgeusia (RD = 8.68%, 95% CI 8.43–8.93%)	A considerable proportion experiences COVID-19 symptoms up to 12 months after infection especially for dysgeusia and dysosmia. Being female or middle-aged increases risks.
Teaima A.A. et al., 2022 [65] Ain Shams, Egypt	Prospective	1031 patients, aged 18–70 (M = 328, F = 703)	From 1 August to 31 October 2020.	Not reported	From 1 week to 6 months after infection.	Clinical history collection.	Dysfunctions occurred after COVID-19 infection were anosmia and ageusia in 50.2%, hyposmia & dysgeusia in 23.3%, anosmia alone in 17.7%.	Most recovery of olfactory/gustatory dysfunction in COVID-19 infection occurs at the first two weeks and is unrelated to patient demographics, treatment or olfactory training.
Titze-de-Almeida R. et al., 2022 [66] Brasília, Brazil	Cohort	236 patients, aged 19–81 (M = 92, F = 144)	From September to December 2020	Moderate-severe	3–8 months after diagnosis	Clinical history collection and completion of questionnaire	Hyposmia (48.3%) and dysgeusia (45.8%) were prevalent symptoms in acute phase whereas there is no statistical significance with LC.	The SARS-CoV-2 infection leads to persistent oral symptoms during LC, in which memory problems may be associated with sleep and depressive complaints.
Travi G. et al., 2021 [67] Milan, Italy	Cohort	901 patients, aged 52–77 (M = 556, F = 345)	From 23 February to 31 May 2020.	Moderate (57.4%), severe (14%), critical (26%)	Not reported	Clinical history collection	One neurological symptom or disease was observed in 30.2% of subjects ranging from dysgeusia/anosmia (9.1%)	Neurologic manifestations in COVID-19 are common but heterogeneous and mortality in subjects with isolated neurologic manifestations seems lower than in those with respiratory symptoms.
Tuter G. et al., 2022 [68] Ankara, Turkey	Cross-sectional	204 patients, aged 53.3 ± 17.8 (M = 76, F = 128)	From February to March 2021	Not reported	Not reported	An online questionnaire of 5 sections and 35 questions.	Xerostomia and dysgeusia were the most common oral manifestations (44.2%, 57.3%) in COVID-19 patients.	SARS-CoV-2 could cause oral manifestations.
Yadav V. et al., 2022 [69] Punjab, India	Prospective	152 patients, aged 43.03 ± 16.10 (M = 78, F = 74)	March 2020	Not reported	4–21 days after diagnosis	Questionnaire of Olfactory Disorders-Negative Statements (sQOD-NS).	Dysgeusia and anosmia were noticed in 20/152 (13.15%).	Olfactory and gustatory dysfunctions are significant part of clinical spectrum of COVID-19 disease In Indian Population.

**Table 4:** **Study Characteristics (*oral mucosal lesions*):** ICU, intensive care unit; RR, relative risk; EM, erythema multiform; ABH, angina bullosa hemorrhagic.

First Author, Year, Reference and Country	Type of Study	Total. *n*. of Patients, Age, Gender	Date Data Collected	COVID-19 Severity and Latency (Days)	Period of Appearance	Diagnostic Method	Results	Conclusions
Al-Magsoosi M.J.N. et al., 2023 [14] Basrah, Iraq	Cross-sectional	574 patients aged 18–79 (M = 196, F = 378)	From October 2021 to April 2022.	Mild–moderate	1–6 months after diagnosis of COVID-19	Questionnaire to collect information from each patient.	Oral ulceration (16.5%)	There is a positive correlation between the incidence of oral signs and symptoms associated with COVID-19 infection and the severity of the infection.
Alhamed S. et al., 2023 [70] Jeddah, Saudi Arabia	Case-control	22 patients, aged 57.9 ± 16.1 (M = 16, F = 6)	From 1 September to 30 October 2021.	Moderate-severe	Not reported	Intra and extra oral objective examination and VAS scale	Candidal infections (68%), oral ulcerations on buccal mucosa, tongue and palatal mucosa (36%), white patches (27.3%) were observed.	Candidal infection and taste disturbance were the most frequent oral lesions in hospital-admitted COVID-19 patients. Other less common manifestations were oral ulcerations.
Amorim Dos Santos J. et al., 2020 [71] Brasilia, Brazil	Case report	1 patient, 67-year-old man	March 2020	Severe	24 days after COVID-19 diagnosis	Intraoral examination, scrape culture.	Recurrent herpes simplex, candidiasis and ulcers on the tongue dorsum.	The importance of the clinical dental examination of patients with infectious diseases in the ICU should be emphasized, considering the need for support, pain control and quality of life.
Batista A.A.F. et al., 2022 [72] Alagoas, Brazil	Case-series	38 patients, aged 58.5–81.3 (M = 12, F = 26)	From 30 July to 30 November 2020	Severe	4–7 days after orotracheal intubation	Intra and extra oral objective examination	Among the patients with oral lesions, ulcerative oral lesions were reported in 14 (87.5%) patients, of which 11 (78.6%) were found on the lips, tongue and palate.	A large proportion of patients with severe COVID-19 develop oral lesions within the first few days of orotracheal intubation.
Binmadi N.O. et al., 2022 [16] Jeddah, Saudi Arabia	Cross-sectional	195 patients, aged 18–70 (M = 48, F = 147)	From October 2021 to March 2022	19% mild, 61% moderate and 19% severe.	1–7 days after the onset of COVID-19 symptoms.	Patient interview and clinical history collection.	Oral ulceration, petechiae, candidiasis, vesiculobullous lesions and erythema migrans. A total of 57 (29%) had oral manifestations; the most common was oral ulcers (11%).	Oral manifestations of COVID-19 were common among female patients and linked to certain general COVID-19 symptoms regarding frequency and extent.
Brandão T.B. et al., 2021 [73] São Paulo, Brazil	Case series	8 patients (M = 5, F = 3) of 28–83 years old	From March to May 2020	Mild-severe	3–10 days after COVID diagnosis	Intraoral examination	Necrosis, hemorrhagic ulcerations with necrotic areas were observed in all patients.	The findings of this case series have highlighted the possible development of oral lesions early in the course of SARS-CoV-2 infection.
Chawla J. et al., 2022 [18] Mangalagiri, India	Cross-sectional	367 patients, aged 18–60 (M = 257, F = 110)	From September to December 2020	Mild–moderate	Not reported	Patient interview, clinical history collection and intra and extra oral objective examination.	Erythema of palate, bilateral buccal mucosa, soft and hard palate and tongue; ulcers of tongue, buccal mucosa, lower labial mucosa and floor of mouth, angular cheilitis and vesicles of palate.	Xerostomia and dysgeusia are the common oral manifestations of COVID-19.
de Paula Eduardo et al., 2022 [74] São Paulo, Brazil	Cohort	472 patients, aged 18–101 (M = 322, F = 150)	From May 2020 to February 2021	Severe	Not reported	Intra and extra oral objective examination and clinical history collection.	In 51.3% alterations in the oral cavity were noted: the most frequent changes were 24.1%, petechiae and varicoses, and 4.4%, dry mouth and sialorrhea.	COVID-19 patients in the intensive care unit often showed dryness in the oral and mucosa oral lesions related to vascular/coagulation disturbances.
El Kady DM et al., 2021 [21] Cairo, Egypt	Cross-sectional	58 patients, aged 18–46 (M = 31, F = 27)	From 15 May to 10 June 2020	Not reported	Not reported	Online questionnaire created by Google Form	Oral ulcers (19.4% in male patients and 14.8% in female patients), tongue redness (3.2% in male patients and 7.4% in female patients)	COVID-19 significantly impacts the oral cavity and salivary glands, as salivary gland-related symptoms and taste disorders are highly prevalent in COVID-19 patients.
El Tantawi M. et al., 2022 [22] Alexandria, Egypt	Cross-sectional	5342 patients, aged 18–23 (M = 2219, F = 3123)	From August 2020 to January 2021	Not reported	Not reported	Questionnaire completed by the patients.	Oral ulcerations were reported in 42.7% of participants.	Dry mouth and changed taste may be used as an indicator for COVID-19 infection in low COVID-19-testing environments.
Elamrousy W.A.H. et al., 2021 [75] Kafrelsheikh, Egypt	Cross-sectional	124 patients, aged 50.32 ± 12.47 (M = 92, F = 32)	From September 2020 to June 2021.	Moderate (41.9%) -severe (58.1%)	Not reported	Clinical history collection and intra and extra oral objective examination.	Oral ulcers represented the most prevalent lesions in the oral cavity in 104 patients (92.8%). Lip, tongue and labial mucosa showed the most common sites for oral ulcers.	The tongue represented the most common site of oral lesions in COVID-19 patients followed by the labial mucosa. No correlation was found between the oral lesions and the drugs used for the treatment of SARS-CoV-2 infection.
Emelyanova N. et al., 2021 [23] Kharkiv, Ukraine	Case-report	38-year-old female patient	September 2020	Not reported	Not reported	Clinical history collection and intra and extra oral objective examination.	Whitish spots, keratosis and desquamation of tongue, exfoliative cheilitis and petechiae.	Importance of a dentist as part of a multidisciplinary team in diagnostics and treatment of COVID-19 was noted.
Fathi Y. et al., 2021 [76] Alborz, Iran	Case report	1 patient, 22-year-old female	Not reported	Not reported	Not reported	Intra and extra oral objective examination	Extensive ulcers in the mouth and crusts on the lips. The diagnosis was of erythema multiform-like.	SARS-CoV-2 could travel to other tissues such as skin and make alterations to the cutaneous immune system, resulting in various manifestations on the skin.
Favia G. et al., 2021 [26] Bari, Italy	Observational	123 patients (F = 53, M = 70), median age of 72 years.	From October to December 2020	Moderate (77%), severe (17%) and critical (6%)	Not reported	Intraoral examination, citology	Geographic tongue (7), fissured tongue (5), ulcerative lesion (65), blisters (33), hyperplasia of papillae (48), angina bullosa (21), candidiasis (18), ulcero-necrotic gingivitis (7), petechiae (14).	This study on a large series highlighted that oral lesion in more than half of cases (65.9%) occurred in the early stage of COVID-19 before the beginning of specific therapies.
Favia G. et al., 2023 [77] Bari, Italy	Cross-sectional	103 patients, aged 69.94 ± 10.99 (M = 55, F = 45)	From January to March 2022	Mild–moderate	Not reported	Intra and extra oral objective examination.	Single and multiple ulcers and multiple petechiae and candidiasis.	Risk of presenting with severe COVID-19 disease was higher in patients who developed oral lesions related to COVID-19 than those with no oral lesions (RR = 7.998, *p* = 0.002).
Ferdeghini C. et al., 2022 [27] Monza, Italy	Observational	58 patients (F = 20, M = 38), 22–92 years old	From April to May 2021	Severe	0–10 days after COVID diagnosis	Intraoral examination	White tongue (22.4%), aphthous lesions (10.3%) and grooves on the tongue (8.6%).	This study therefore seems to demonstrate a possible correlation between the alterations found in patients hospitalized for COVID-19 and the infection itself by the virus.
Fernandes T.J. et al., 2023 [28] Rio de Janeiro, Brazil	Cross-sectional	136 patients, aged 37 ± 14.13 (M = 57, F = 79) divided into 2 groups: rRT-PCR-positive and rRT-PCR-negative patients.	From October 2020 to September 2021	Not reported	Not reported	Intra and extra oral objective examination and intervist by dentist.	Mouth ulcers and petechiae were reported in both groups without significant differences.	A high prevalence of oral manifestations was observed in symptomatic patients with suspected or confirmed COVID-19.
Ferreira M.D. et al., 2023 [78] Ponta Grossa, Brazil	Case-control	112 patients divided into 2 groups: 69 COVID-19 positive and 43 COVID-19 negative patients; aged 56.3 ± 14.9 (M = 67, F = 45).	Not reported	Severe	Not reported	Intra and extra oral objective examination.	Hemorrhagic ulcers (*p* = 0.047), pressure ulcers (*p* < 0.001) and angular cheilitis (*p* = 0.035) were significantly associated with hospitalized patients with COVID-19 positive.	There may be an association between hospitalization for COVID-19 and the development of oral changes, including bleeding ulcers, pressure ulcers. and angular cheilitis.
Fidan V. et al., 2021 [79] Eskisehir, Turkey	Observational	74 patients (F = 33.8%, M = 66.2%), aged 49.3 ± 7.2 years	From April to October 2020	Not reported	Not reported	Intraoral examination	Aphthous-like ulcer (46.6%) Erythema (32.8%) Lichen planus (20.6%).	The oral circumstances confronted by this survey and other published studies fortifies the theory which they are enthusiastically implicative of secondary lesions occurring from the impairment of systemic vigor or appropriate to therapies for COVID-19.
Ganesan A. et al., 2022 [30] Rajasthan, India	Cross-sectional	500 patients, aged 53.46 ± 17.50 (M = 367, F = 133)	From 18 October to 7 November 2020	Mild	Not reported	Intra and extra oral objective examination	15.4% of patients were found to have oral findings like erythema, ulcers, depapillation of tongue atrophic glossitis, candida-like lesions. There was a statistically significant correlation between oral manifestations and disease severity.	COVID-19 is found to effect oral health with greater probability in patients with severe diseases.
Glavina A. et al., 2024 [5] Split, Croatia	Case series	15 patients aged 24–85 (M = 6, F = 9)	From November 2020 to January 2024	Mild-severe 3–60 days	Not reported	Intra and extra oral objective examination	Hyperplasia, erosion and, erythema on gingiva, buccal mucosa, tongue and hard palate occurred in middle-aged patients, with an equal distribution by sex.	Oral lesions presented in a mild form and did not correlate with the severity of the clinical picture of COVID-19.
Goel S. et al., 2022 [80] Utar Pradesh, India	Case series	5 patients, aged 53–59 (M = 3, F = 2)	From January to December 2021	Mild–moderate	Not reported	Intra and extra oral objective examination.	Single blood-filled bullae measuring 1 to 2 cm in diameter on buccal mucosa, palate and lower labial mucosa.	Classical clinical features lead to the diagnosis of angina bullosa hemorrhagic in all the 5 patients.
Gogotishvili M. et al., 2024 [32] Batumi, Georgia	Cohort	55 patients, aged 18 to 89 (M = 30, F = 25)	Not reported (during the COVID-19 pandemic)	Mild–moderate	Not reported	Intra and extra oral objective examination.	Angular cheilitis, lichenoid lesions, candidiasis, ulcers enanthems on buccal mucosa and hard palate and geographic tongue and caviar tongue 40% of the patients had at least 1 oral lesion. The most common lesions were candidiasis and ulcers	Oral mucosal alterations and lesions were prevalent in this series of COVID-19 patients.
Hussain S. et al., 2023 [35] Peshawar, Pakistan	Cross-sectional	117 patients, aged 15–70 (M = 85, F = 32)	From 1 October to 30 January 2021	Not reported	10.62 ± 6.19 days after infection	Clinical history collection and intra and extra oral objective examination.	Oral ulcers on lips or on corners of the mouth were experienced by 10.3% (*n* = 12), angular cheilitis 6% (*n* = 7), white lesions 0.9% (*n* = 1).	Oral manifestations were observed in 56% of PCR- confirmed COVID-19-positive patients, with taste alterations being the most common at 49.6%.
Jafarzadeh J. et al., 2023 [81] Babol, Iran	Case-report	1 female patient, 83-year-old	June 2020	Severe	4–7 weeks	Intra and extra oral objective examination and cultural examination.	Candidiasis on tongue and buccal mucosa and fissured tongue.	Risk of fungal infections, such as Candida glabrata seems to be high in patients with severe COVID-19, mainly affecting the oral mucosa.
Johansson A.K. et al., 2023 [37] Bergen, Norway	Cohort	8203 patients, aged 89–90 (M = 3662, F = 4541)	April 2022	Mild–moderate	16 months (range 5–28 months).	Oslo COVID-19 questionnaire.	For ulcer/blisters in the mouth and on the lips *p*-value was not significant.	Long-COVID general and orofacial symptoms are common among older elderly COVID-19 survivors.
Kitakawa D. et al., 2020 [82] Mogi das Cruzes, Brazil	Case report	1 patient, 20-year-old female	April 2020	Not reported	At the same time of COVID diagnosis.	Intraoral examination	Herpes simplex	Could the clinical manifestation of lesions, compatible with some viral infections such as recurrent herpes, be truly associated with COVID-19?
Lee H. et al., 2023 [83] California, Los Angeles USA	Case-report	40-year-old male patient	From April 2020 to June 2023	Mild–moderate	2 weeks after COVID-19 infection. The oral lesions also appeared after 3 years after a COVID-19 infection 1–2 weeks prior.	Intra and extra oral objective examination.	Herpes viral gingivosto-mastitis, herpes-associated erythema multiforme, candidiasis, bilateral ulcers and vesicles in the buccal mucosa, palate, throat, attached gingival and inner and outer lips and erythematous ulcerous lesions in the anterior vestibule and floor of the mouth along with blood-crusted lips.	The presence and reactivation of oral lesions in a COVID-19 recovered patient could be related to COVID-19s profound role in immune dysregulation or related therapies.
Limongelli L. et al., 2023 [84] Bari, Italy	Observational	6 patients, aged 9–65 (M = 4, F = 2)	From September 2021 to September 2022	Mild–moderate	10.3 weeks after infection	Intra and extra oral objective examination and histopathological examination.	Buccal mucosa, lower and upper lip, vermilion ulceration were observed in all patients.	SARS-CoV-2 can persist, as for other organs/systems, also in the oral epithelium/mucosa after the acute phase and can be responsible for lesions.
Maden C.L. et al., 2021 [85] London, UK	Case report	1 patient, 18-year-old man	Not reported	Severe	3 weeks after diagnosis of COVID-19.	Intra and extra oral objective examination and incisional biopsy.	Oral ulcerations of the lips. The diagnosis was of erythema multiform-like.	This case highlights the consideration of COVID-19 associated EM as a diagnosis in a patient presenting with membranous conjunctivitis and mucosal ulceration.
Mahmoud M. et al., 2022 [86] Cairo, Egypt	Case-series	5 patients, aged 3–32 (M = 1; F = 4)	September 2020	Not reported	3–12 days after diagnosis.	Clinical history collection and intra and extra oral objective examination.	Glossitis, candidiasis, erythematous erosion on the side of the anterior third of the tongue, aphthous ulceration.	Oral examination is mandatory in cases with suspected or confirmed COVID-19 infection.
Metin N. et al., 2024 [43] Erzurm, Turkey	Cross-sectional	383 patients, aged 39 ± 12 (M = 214, F = 168)	From 1 January to 30 June 2020	Not reported	1–3 months	Intra and extra oral objective examination and PCR test.	Dry mouth, microvesicles of buccal mucosa, angular cheilitis, oral aphthae and scrotal tongue were significantly higher in patients with positive PCR tests.	There is a significant correlation between COVID-19 infection, PCR positivity and the numerous manifestations of oral lesion.
Mohammed F. et al., 2023 [46] Dammam, Saudi Arabia	Observational	179 patients, aged 10–51 (M = 102, F = 77)	From August to December 2020	Mild–moderate	1–7 weeks	Questionnaire by QuestionPro software.	Ulcers (8.2%), vesicles (7.0%) and papules (3.2%) on lips, buccal mucosa, hard and soft palate, retromolar area, posterior tongue, tonsil and uvula.	The results of this study indicate that the presence of oral mucosal lesions and dysgeusia or dry mouth and a burning sensation, along with COVID-19 generic symptoms, should be regarded as suggestive but not definite markers of COVID-19.
Muthyam A.K. et al., 2022 [48] Telangana, India	Cross-sectional	100 patients, aged 20–73 (M = 51, F = 49)	Not reported	Mild–moderate	2 weeks to 3 months after diagnosis of COVID-19.	Questionnaire: a close-ended, validated, questionnaire containing 25 questions.	Oral ulcerations (10%) on buccal mucosa and tongue.	Xerostomia, frequent aphthous ulcers, swallowing difficulty and burning mouth were the most frequently encountered symptoms in study subjects during the disease and post recovery.
Naser A.I. et al., 2021 [49] Mosul, Iraq	Prospective	338 patients (F = 138, M = 200)	From August 2020 to March 2021	Not reported	Not reported	Extra and intraoral examination and radiographic evaluation (if needed).	White coat of the tongue (31.6), white coat of the gingiva, cheek (22.4%) and palate (15.6%), red discoloration in the oral cavity (4.7%), blue discoloration of the tongue and lips (6.8%), black discoloration of the oral cavity (4.7%), apthous ulceration anywhere in the oral cavity (9.1%).	Most of the intraoral and extraoral lesions are temporary and disappear with appropriate treatment in COVID-19 patients, so early treatment is quite important to decrease complications.
Natto Z.S. et al., 2021 [50] Jeddah, Saudi Arabia	Cross-sectional	109 patients, aged 39.3 ± 12.4 (M = 76, F = 36)	From 28 July to 5 October 2020	Mild	2–15 days after infection	Questionnaire and clinical examination.	Ulcers and blisters (6.4%), coated tongue (7.3%), erythema and desquamated. The most affected site wee: dorsum of tongue, vestibules, buccal mucosa, lips and gingiva.	Oral examinations of COVID-19 patients should be conducted as part of routine examinations to investigate any possible correlation between the disease and the oral cavity.
Nayak P. et al., 2023 [87] Uttar Pradesh, India	Case-series	35 and 41-year-old female patients	Not reported	Not reported	4–6 weeks after diagnosis of COVID-19	Intra and extra oral objective examination and histopathological examination	Dark, reddish brown vescicle over the right lateral border of tongue and over the soft palate.	The diagnosis was of angina bullosa hemorrhagic. Since ABH has been reported in COVID-19, it is plausible that some of the mechanisms underlying the pathogenesis of oral manifestations may explain the pathogenesis of ABH.
Nejabi M.B. et al., 2021 [88] Guangzhou, China	Case-report	62-year-old male patient	August 2020	Moderate	Not reported	Clinical history collection and intra and extra oral objective examination.	Erosive ulcers on the dorsal surface of tongue.	Patients with suspected or confirmed SARS-CoV-2 should be screened for symptoms and physical findings in the oral mucosa.
Nikalje M.R. et al., 2021 [51] Maharashtra, India	Observational	201 patients (M = 150, F = 51)	From June to September 2020	Not reported	Not reported	Routine screening procedure.	Oral ulcerations (1.5%).	Oral ulcerations are related to COVID-19 severity.
Ortiz G. et al., 2022 [89] Pennsylvania, USA	Case-report	16-year-old male patient	Not reported	Severe	Not reported	Clinical history collection, intra and extra oral objective examination and biopsy.	Papules and plaques on tongue and lips. The diagnosis was of reactive infectious mucocutaneous eruption characterized by significant mucositis involving the oral and genital mucosa.	Immunohistochemical investigations of RIME are encouraged to determine if this is a unique histopathologic presentation, possibly related to COVID-19.
Palaia G. et al., 2022 [90] Caserta, Italy	Case-report	30-year-old female patient	Not reported	Not reported	7 days before the specialist visit	Patient interview and intra and extra oral objective examination.	Oral ulcerations- aphthous like in the vermillion lips, oral vesicle in buccal and cheek mucosa, oral ulcer in palate and blisters in the dorsum of the tongue.	Confirming the literature studies, erythema multiforme is an early disease associated with COVID-19 infection.
Patel D. et al., 2024 [54] Portsmouth, UK	Cross-sectional	104 patients, aged 18–79 (M = 8, F = 93, other = 3)	From February to May 2022	Mild–moderate	From 1–7 days to more than 3 months.	Self-reported online questionnaire and healthcare professionals ‘semi-structured interviews.	Mouth ulcer, erosion and herpetiformis (35.6%), white/red plaques (18.3%), loss of papillae on tongue surface (1.9%).	Healthcare professionals have observed oral manifestations in individuals diagnosed with long-COVID.
Rueda C.A.C. et al., 2023 [91] Mexico City, Mexico	Case series	10 patients, aged 50–75 (M = 9; F = 1)	From March to May 2021	Severe	8–32 days after COVID-19 infection.	Intra and extra oral objective examination and histopathological examination.	Bleeding and herpetiform ulcers on lips, corner lips, tongue, gum and inner cheeks.	A direct connection between the virus and oral manifestations cannot be concluded.
Saleh W. et al., 2021 [92] Mansoura, Egypt	Case-report	63-year-old male patient	April 2021	Not reported	Not reported	Intra and extra oral objective examination and biopsy.	Painful erosive areas of the buccal mucosa and the dorsal surface of the tongue. The lesion of the left buccal mucosa shows a central erosive area surrounded by white radiating striae from the periphery of the lesion.	Our case suggests the possible association between COVID-19 and oral lichen planus.
Sircar K. et al., 2022 [93] Uttar Pradesh, India	Case series	4 male patients of 34–84 years old	Not reported	Moderate-severe	3–10 days after COVID-19 diagnosis.	Intraoral examination	Multiple small ulcers in the soft palate mucosa; large ulcers on the right lateral border of the tongue; multiple depapillated erythematous areas on the tongue; multiple of upper and lower lips and hemorrhagic encrustation along with angular cheilitis.	Exists a reasonable connotation between oral health status and severity of COVID-19 symptoms.
Dalipi S.Z. et al., 2021 [19] Prishtina, Kosovo	Case report	17-year-old male patient	Not reported	Moderate-severe	2 week after diagnosis	Clinical history collection and intra and extra oral objective examination.	Bullous and erosive erythematous lesions on the lips, vesiculobullous/macular lesions on the oral mucosa.	The diagnosis was of exudative erythema multiforme.
Soares C.D. et al., 2022 [94] São Paulo, Brazil	Retrospective	14 patients, aged 23–88 (M = 10, F = 4)	From May to September 2021	Moderate	21–28 days after infection	Patient interview, clinical history collection and intra and extra oral objective examination.	8 had ulcerative lesions and petechiae only in the palate (57.1%), 4 had tongue lesions and 2 presented ulcerative lesions in either the lip or palate (14.3%).	The detection of viral proteins in the oral mucosa and the presence of thrombotic vessels and hemorrhage elucidate COVID-19 pathogenesis in the oral mucosa.
Subramaniam T. et al., 2021 [95] Maharashtra, India	Observational	713 patients, aged 18–71 (M = 416, F = 297)	From April 2020 to June 2020	Moderate	Not reported	Clinical history collection and intra and extra oral objective examination.	9 patients reported oral discomfort due to varied forms of oral lesions ranging from herpes simplex ulcers to angular cheilitis (1.26%).	This study supports the hypothesis that oral manifestations in patients diagnosed with COVID-19 could be secondary lesions resulting from local irritants or from the deterioration of systemic health.
Tavakoli F. et al., 2022 [96] Shiraz, Iran	Cross-sectional	55 patients (F = 22, M = 33) 38.52 ± 10.466 years old	From January 2021 to March 2021	Mild	4.29 day after COVID diagnosis.	Intraoral examination.	Recurrent aphthous stomatitis (1.8%), recurrent herpes (9.1%).	The oral symptoms in patients with COVID-19 infection might be the earliest symptom even if the patient is asymptomatic and he is not aware of his sickness.
Tuter G. et al., 2022 [68] Ankara, Turkey	Cross-sectional	204 patients, aged 53.3 ± 17.8 (M = 76, F = 128)	From February 2021 to March 2021	Not reported	Not reported	An online questionnaire of 5 sections and 35 questions.	The most common oral manifestations were oral lesions (22.4%) such as oral ulcer (14.5%) and tongue lesions (6.7%).	SARS-CoV-2 could cause oral manifestations.
Santos N.M.V.D. et al., 2022 [97] Recife PE, Brazil	Retrospective	89 patients, aged 0–12 (M = 54; F = 35)	From March to August 2020	Moderate-severe	Not reported	Intra and extra oral objective examination	Of 89 children, 20.2% had oral manifestations and mucositis was the most prevalent lesion (12.4%).	This study reveals that children with SARS-CoV-2 with oral lesions stay longer in hospital and have more severe conditions.
Villanueva-Sánchez F.et al, 2023 [98] New Mexico, Mexico	Case report	2 male patients of 67 and 47 years old	From November 2020 to November 2021	Not reported	1: 1 month after COVID-19diagnosis. 2: at the same time of COVID-19diagnosis.	1: Intraoral examination. 2: Intraoral examination and histological examination.	1: two erosive lesions on the lateral edges of the tongue. 2: vesiculobullous oral lesions.	Exists a reasonable connotation between oral health status and severity of COVID-19 symptoms.
Yeom J. et al., 2023 [99] New York, USA	Case series	3 patients, aged 70–83	Not reported	Severe	11–22 days after infection.	Intra and extra oral objective examination and histopathological examination.	Stellate, shaped ulcerations on ventral, dorsal and lateral tongue and labial mucosa and pseudomembranous candidiasis.	Awareness of the possible atypical presentations of herpes in COVID-19 patients undergoing systemic therapy will allow for their timely diagnosis and appropriate treatment.

**Table 5:** **Study Characteristics (*osteonecrosis of the jaws*):** CBCT, cone beam computed tomography; OPG, orthopantomogram; PAS, periodic acid-Schiff; GMS, Grocott methenamine–silver; CAM, complementary and alternative medicine.

First Author, Year, Reference and Country	Type of Study	Total. *n*. of Patients, Age, Gender	Date Data Collected	COVID-19 Severity and Latency (Days)	Period of Appearance	Diagnostic Method	Results	Conclusions
Al-Mahalawy H. et al., 2022 [100] Alexandria, Egypt	Case-series	12 patients aged 56.1 ± 9.65 (M = 5, F = 7)	From January to August 2021	Moderate-severe	3–12 weeks from the day of the negative PCR test	Intra and extra oral objective examination and CBCT imaging.	Maxillary osteonecrosis.	Post-COVID-related osteonecrosis of the jaw could be now considered as one of the potential post-COVID-19 oral and maxillofacial complications that occurs unprovokedly and mainly in the maxilla.
Ansari S. et al., 2023 [101] Belagavi, India	Observational	76 patients, aged 32–75 (M = 72, F = 4)	From March to December 2021	Moderate-severe	Not reported	Intra and extra oral objective examination, CBCT imagin and fungal culture.	Among 76 individuals with COVID-19-associated rhinomaxillary mucormycosis diabetes mellitus was present in 93.42% of cases. Almost all patients received corticosteroids during COVID-19 treatment.	Early signs and oral manifestations of rhinomaxillary mucormycosis play a pivotal role in the early diagnosis and prompt treatment to reduce mortality and morbidity in COVID-19 associated- rhinomaxillary mucormycosis patients.
Arshad W. et al., 2022 [102] Islamabad, Pakistan	Case report	1 patient, 56-year-old male	Not reported	Not reported	4 months after diagnosis of COVID-19.	Intraoral examination, CT, histopathologic evaluation.	Actinomycotic osteomyelitis of maxillae.	Positive causal association between COVID-19 and actinomycosis.
Arthanari K.K. et al., 2021 [103] Nadu, India	Case-report	A 67-year-old male patient	Not reported	Not reported	Not reported	Intra and extra oral objective examination, CBCT imaging and fungal culture.	Maxillary osteonecrosis with fungal infection (mucormycosis).	Due to the rapid increase in cases of mucormycosis in COVID-19, there is an immense need to improve awareness among treating physicians and dentists about early diagnosis and treatment.
Fakhar M. et al., 2022 [104] Sari, Iran	Case-series	2-man patients of 35 and 40-year-old	Not reported	Moderate-severe	20–40 days after infection.	Intra and extra oral objective examination, CBCT imaging and OPG.	Maxillary osteonecrosis with necro- inflammation and mucormycosis.	Corticosteroids and tocilizumab regimens used alone or in combination for the treatment of progressive COVID-19pneumonia should be aware of the potential risks of invasive maxillofacial fun- gal infections and jaw osteonecrosis.
Kulkarni D. et al., 2024 [105] Maharashtra, India	Case-report	41-year-old male patient	July 2022	Moderate - severe	Not reported	Intra and extra oral objective examination, CBCT imaging, OPG and histopathological analysis.	Maxillary mucormycosis.	Coinfections of HIV/TB, COVID-19 and mucormycosis continue to pose a threat to healthcare providers and immunocompromised individuals, representing a new and global pathogenic scenario.
Romano A. et al., 2024 [106] Naples, Italy	Case report	1 patient, 74-year-old male patient	November 2021	Severe	3 months after diagnosis of COVID-19.	Intraoral and extraoral examination, CT scan, histopathologic evaluation.	Left maxillary oro-antral fistula with purulent exudate and a diffuse necrotic. The diagnosis was of actinomycotic osteonecrosis of the upper maxilla with mucormycosis.	SARS-CoV-2 infection may amplify the risk of superinfections by opportunistic pathogens like Aspergillus and Mucor.
Roopa R. et al., 2021 [107] Chennai, IND	Case-report	59-year-old male patient	April 2021	Severe	Not reported	Intra and extra oral objective examination, CBCT imaging, OPG and histopathological analysis.	Mazillary, zygomatico-maxillary process and hard palate osteonecrosis with fungal infection (mucormycosis).	Massive use of antibiotics, antibodies, steroids during the management of COVID-19 have caused increase in several bacterial, fungal and viral infections.
Sevagaperumal A. et al., 2024 [108] Tamil Nadu, India	Case Series	10 patients, aged 31–82 (M = 6, F = 4)	Not reported	Not reported	Not reported	Intra and extra oral objective examination, CBCT imaging, OPG and histopathological analysis.	Maxilla and mid-palate osteonecrosis with fungal infection (mucormycosis).	The incidence of mucormycosis has increased in immunocompetent patients with emergence of COVID-19 infection.
Slavkova N. et al., 2022 [109] Sofia, Bulgaria	Case-report	70-year-old male patient	Not reported	Severe	1 month after diagnosis of COVID-19	Intra and extra oral objective examination and CBCT imaging.	Osteonecrosis of the right maxillary and zygomatic bone with a pathologic communication connecting the maxillary sinus and the oral cavity.	Serious complications have been observed in the facial area due to COVID-19 disease and/or its treatment, from minor changes in the oral cavity to osteonecrosis of the jaw.
Sood A. et al., 2023 [110] Delhi, India	Case-series	4 male patients of 28–65 years old	Not reported	Moderate-severe	15 days–2 months after infection.	Intra and extra oral objective examination, CBCT imaging, OPG and histopathological analysis.	Maxillary posterior and body-angle-ramus of mandible osteonecrosis.	Steroid therapy, along with COVID-19-induced impairment in micro- circulation, may have complementary role in producing these complications of avascular necrosis in susceptible regions of the body.
Urias-Barreras C.M. et al., 2023 [111] Sinaloa, Mexico	Case series	20 patients, aged 59.7 ± 14.0 (M = 11, F = 9)	2020–2022	Moderate-severe	1.5–6 months after diagnosis of COVID-19.	Intra and extra oral objective examination, CBCT imaging and histomorphology evaluation by PAS and GMS.	Osteonecrosis of the maxilla with exposed necrotic bone, tooth mobility and halitosis. Unilateral maxillary sinus involvement (30%) and bilateral maxillary sinus involvement (15%).	It is essential to consider the association of osteonecrosis of the jaw in post-COVID-19 patients, with aspergillosis and mucormycosis, for early diagnosis and appropriate treatment.
Vasanthi V. et al., 2024 [112] Tamilnadu, India	Case report	1 patient, 55-year-old female	Not reported	Not reported	2 months after diagnosis of COVID-19	Intraoral examination, CT, histopathologic evaluation.	Actinomycotic osteonecrosis of the maxilla.	COVID-19 infection further causes immune dysregulation, thereby increasing the susceptibility to opportunistic infections.
Vijapur M.M. et al., 2022 [113] Karnataka, India	Observational	60 patients, aged 25–76 (M = 36; F = 15)	From 1 June 2021, to 31 September 2021	Moderate	37.93 ± 25.41 days after infection	Intra and extra oral objective examination, CBCT imaging, OPG and fungal culture.	About 50% of subjects presented with “Rhino orbital” type of Mucormycosis with maxillary osteonecrosis Palatal discoloration and erosion were the most common oral manifestation.	The study indicates that diabetes mellitus is the most associated comorbidity in CAM patients.

## Data Availability

The original contributions presented in this study are included in the article/Supplementary Materials. Further inquiries can be directed to the corresponding author.

## Supplementary Materials

The following supporting information can be downloaded at: https://www.mdpi.com/article/10.3390/jcm14041267/s1, Table S1: Assessment of quality and risk of bias for case-report; Table S2: Assessment of quality and risk of bias for case-control and retrospective studies; Table S3: Assessment of quality and risk of bias for case-series; Table S4: Assessment of quality and risk of bias for cohort and prospective studies; Table S5: Assessment of quality and risk of bias for cross-sectional and observational studies.
