# Supplementary material for: Unveiling the Oral Lesions, Dysgeusia and Osteonecrosis Related to COVID-19: A Comprehensive Systematic Review
Source: J Clin Med. 2025 Feb 14;14(4):1267. doi: 10.3390/jcm14041267 (PMC11856591; doi:10.3390/jcm14041267)
Supplement: Supplementary file 1 [file jcm-14-01267-s001.zip › jcm-3482749-supplementary.pdf]

STUDY	Were patient's demographic characteristics clearly described?	Was the patient's history clearly described and presented as a timeline?	Was the current clinical condition of the patient on presentation clearly described?	Were diagnostic tests or assessment methods and the results clearly described?	Was the intervention(s) or treatment procedure(s) clearly described?	Was the post-intervention clinical condition clearly described?	Were adverse events (harms) or unanticipated events identified and described?	Does the case report provide takeaway lessons?	Overall appraisal
**Amorim Dos Santos J. et al, 2020 [71]**	Y	U	Y	U	Y	Y	N	Y	**5/8 moderate**
**Arshad W. et al, 2022 [102]**	Y	U	Y	U	Y	Y	N	Y	**5/8 moderate**
**Arthanari K.K. et al, 2021 [103]**	Y	Y	Y	U	Y	Y	N	Y	**6/8 low**
**Dalipi S.Z. et al, 2021 [19]**	Y	Y	Y	Y	Y	Y	N	Y	**7/8 low**
**Eita A.A.B. et al, 2021 [20]**	Y	Y	Y	U	Y	Y	U	Y	**6/8 low**
**Emelyanova N. et al, 2021 [23]**	Y	Y	N	Y	U	Y	N	Y	**5/8 moderate**
**Fathi Y. et al, 2021 [76]**	Y	Y	U	Y	Y	Y	U	Y	**6/8 low**
**Jafarzadeh J. et al, 2023 [81]**	Y	N	N	Y	Y	U	N	Y	**5/8 moderate**
**Kajita M. et al, 2021 [38]**	Y	Y	N	Y	N	Y	N	Y	**5/8 moderate**
**Kitakawa D. et al, 2020 [82]**	Y	N	U	Y	Y	U	U	Y	**4/8 moderate**

**Kulkarni D. et al, 2024 [105]**	Y	N	U	Y	Y	U	U	Y	**4/8 moderate**
**Lee H. et al, 2023 [83]**	Y	Y	Y	Y	Y	U	U	Y	**6/8 low**
**Maden C.L. et al, 2021 [85]**	Y	Y	Y	U	Y	U	U	Y	**5/8 moderate**
**Nejabi M.B. et al, 2021 [88]**	Y	Y	Y	U	Y	Y	U	Y	**6/8 low**
**Ortiz G. et al, 2022 [89]**	Y	Y	Y	U	Y	Y	U	Y	**6/8 low**
**Palaia G. et al, 2022 [90]**	Y	Y	Y	Y	Y	Y	U	Y	**7/8 low**
**Romano A. et al, 2024 [106]**	Y	Y	Y	Y	Y	Y	Y	Y	**8/8 low**
**Roopa R. et al, 2021 [109]**	Y	Y	Y	Y	Y	Y	U	Y	**7/8 low**
**Saleh W. et al, 2021 [92]**	Y	Y	Y	N	Y	Y	U	Y	**6/8 low**
**Sequeria Rodriguez P. et al, 2023 [60]**	Y	N	N	Y	Y	N	U	Y	**4/8 moderate**
**Slavkova N. et al, 2022 [109]**	Y	Y	Y	Y	Y	Y	U	Y	**7/8 low**
**Vasanthi V. et al, 2024 [112]**	Y	Y	Y	U	Y	Y	N	Y	**6/8 low**
**Villanueva-Sánchez F. et al, 2023 [98]**	Y	Y	Y	U	Y	Y	N	Y	**6/8 low**

Table S1: Assessment of quality and risk of bias for case-report studies included in the systematic review. Each domain was satisfied (Y), not satisfied (N), unclear (U), or not applicable (N/A) according to the Joanna Briggs Institute Critical Appraisal tool.

STUDY	Were the groups comparable other than the presence of disease in cases or the absence of disease in controls?	Were cases and controls matched appropriately?	Were the same criteria used for identification of cases and controls?	Was exposure measured in a standard, valid and reliable way?	Was exposure measured in the same way for cases and controls?	Were confounding factors identified?	Were strategies to deal with confounding factors stated?	Were outcomes assessed in a standard, valid and reliable way for cases and controls?	Was the exposure period of interest long enough to be meaningful?	Was appropriate statistical analysis used?	Overall appraisal
**Alhamed S. et al, 2023 [70]**	Y	Y	Y	Y	Y	U	U	Y	U	Y	**7/10 low**
**Falaki M. et al, 2022 [24]**	Y	Y	Y	U	Y	N	N	Y	U	Y	**6/10 moderate**
**Ferreira M.D. et al, 2023 [78]**	Y	Y	Y	Y	U	N	N	Y	U	Y	**6/10 moderate**
**Gençeli M. et al, 2022 [31]**	Y	NA	NA	U	U	Y	U	U	U	Y	**3/10 high**
**Klopfenstein T. et al, 2020 [39]**	Y	U	U	Y	U	U	U	Y	Y	Y	**5/10 moderate**
**Sakurada Y. et al, 2022 [57]**	Y	NA	U	Y	Y	Y	N	Y	N	Y	**6/10 moderate**
**Santos N.M.V.D. et al, 2022 [97]**	Y	NA	U	Y	U	U	U	Y	N	Y	**4/10 high**
**Soares C.D. et al, 2022 [94]**	Y	NA	NA	Y	N	Y	N	NA	N	Y	**4/10 high**

Table S2: Assessment of quality and risk of bias for case-control and retrospective studies included in the systematic review. Each domain was satisfied (Y), not satisfied (N), unclear (U), or not applicable (N/A) according to the Joanna Briggs Institute Critical Appraisal tool.

STUDY	Were there clear criteria for inclusion in the case series?	Was the condition measured in a standard, reliable way for all participants included in the case series?	Were valid methods used for identification of the condition for all participants included in the case series?	Did the case series have consecutive inclusion of participants?	Did the case series have complete inclusion of participants?	Was there clear reporting of the demographics of the participants in the study?	Was there clear reporting of clinical information of the participants?	Were the outcomes or follow up results of cases clearly reported?	Was there clear reporting of the presenting site(s)/clinic(s) demographic information?	Was statistical analysis appropriate?	Overall risk of bias
**Al-Mahalawy H. et al, 2022 [100]**	Y	U	Y	U	Y	Y	Y	Y	Y	Y	**8/10 low**
**Batista A.A.F. et al, 2022 [72]**	Y	Y	Y	N	N	Y	Y	U	U	N	**5/10 moderate**
**Brandão T.B. et al, 2021 [73]**	Y	Y	Y	U	U	Y	Y	Y	Y	N	**7/10 low**
**Fakhar M. et al, 2022 [104]**	N	Y	Y	U	U	N	Y	Y	U	U	**4/10 high**
**Glavina A. et al, 2024 [5]**	N	Y	Y	N	N	Y	Y	Y	Y	U	**6/10 moderate**
**Goel S. et al,2022 [80]**	N	U	Y	U	U	N	Y	U	Y	N	**3/10 high**
**Mahmoud M. et al, 2022 [86]**	Y	U	Y	Y	U	Y	U	N	Y	N	**5/10 moderate**
**Nayak P. et al, 2023 [87]**	Y	N	Y	U	U	Y	Y	Y	Y	N	**6/10 moderate**
**Rueda C.A.C. et al, 2023 [91]**	Y	Y	Y	N	Y	Y	Y	Y	U	N	**7/10 low**
**Sevagaperumal A. et al, 2024 [108]**	U	Y	Y	U	U	Y	Y	U	Y	N	**5/10 moderate**
**Sircar K. et al, 2022 [93]**	Y	Y	Y	N	N	Y	Y	N	Y	N	**6/10 moderate**

**Skrypnikova T. et al, 2022 [63]**	Y	Y	U	U	Y	Y	Y	Y	Y	Y	**8/10 low**
**Sood A. et al, 2023 [110]**	Y	Y	Y	U	U	Y	Y	Y	Y	N	**7/10 low**
**Urias-Barreras C.M. et al, 2023 [111]**	U	Y	Y	U	U	Y	Y	N	Y	N	**5/10 moderate**
**Yeom J. et al, 2023 [99]**	N	N	Y	U	U	Y	Y	Y	U	N	**4/10 high**

Table S3 : Assessment of quality and risk of bias for case-series studies included in the systematic review. Each domain was satisfied (Y), not satisfied (N), unclear (U), or not applicable (N/A) according to the Joanna Briggs Institute Critical Appraisal tool.

STUDY	Were the two groups similar and recruited from the same population?	Were the exposures measured similarly to assign people to both exposed and unexposed groups?	Was the exposure measured in a valid and reliable way?	Were confounding factors identified?	Were strategies to deal with confounding factors stated?	Were the groups/participants free of the outcome at the start of the study (or at the moment of exposure)?	Were the outcomes measured in a valid and reliable way?	Was the follow up time reported and sufficient to be long enough for outcomes to occur?	Was follow up complete, and if not, were the reasons to loss to follow up described and explored?	Were strategies to address incomplete follow up utilized?	Was appropriate statistical analysis used?	Overall appraisal
**Borah H. et al, 2022 [17]**	NA	NA	Y	N	N	Y	Y	U	U	N	N	**3/11 high**
**de Paula Eduardo et al, 2022 [74]**	NA	NA	Y	Y	Y	U	Y	Y	U	Y	Y	**7/11 low**
**Fantozzi P.J. et al, 2020 [25]**	NA	NA	Y	Y	Y	U	Y	Y	U	Y	Y	**7/11 low**
**Gogotishvili M. et al, 2024 [32]**	NA	NA	Y	N	N	U	Y	U	U	N	N	**2/11 high**
**Haran J.P. et al, 2021 [34]**	NA	NA	Y	N	N	U	Y	U	U	N	N	**2/11 high**
**Johansson A.K. et al, 2023 [37]**	Y	Y	Y	N	N	Y	Y	Y	U	N	Y	**6/11 moderate**
**Köseoğlu Toksoy C. et al, 2021 [40]**	Y	Y	Y	U	U	Y	Y	Y	N	N	Y	**7/11 low**
**Kumar L. et al, 2021 [41]**	NA	NA	Y	U	U	Y	Y	U	U	U	U	**3/11 high**
**Mizrahi B. et al, 2023 [45]**	Y	Y	Y	U	U	Y	Y	Y	Y	N	Y	**8/11 low**
**Munsch N. et al, 2022 [47]**	Y	Y	Y	Y	U	Y	U	N	N	N	Y	**6/11 moderate**

**Naser A.I. et al, 2021 [49]**	NA	Y	Y	U	U	Y	Y	Y	Y	U	Y	**7/11 low**
**Otsuka Y. et al, 2021 [52]**	NA	NA	Y	U	U	Y	Y	U	U	U	U	**3/11 high**
**Samaranayake L.P. et al, 2020 [58]**	U	Y	Y	Y	U	Y	Y	N	N	N	Y	**6/11 moderate**
**Schambeck S.E et al, 2021 [59]**	Y	Y	Y	U	U	U	Y	Y	NA	NA	N	**5/11 high**
**Teaima A.A. et al, 2022 [65]**	NA	NA	Y	Y	N	Y	Y	Y	Y	U	Y	**7/11 low**
**Titze-de-Almeida R. et al, 2022 [66]**	Y	Y	Y	Y	U	U	Y	Y	N	N	Y	**6/11 moderate**
**Travi G. et al, 2021 [67]**	Y	Y	Y	Y	U	Y	Y	Y	Y	N	Y	**9/11 low**
**Yadav V. et al, 2022 [69]**	NA	NA	Y	N	N	Y	Y	Y	U	U	Y	**5/11 high**

Table S4: Assessment of quality and risk of bias for cohort and prospective studies included in the systematic review. Each domain was satisfied (Y), not satisfied (N), unclear (U), or not applicable (N/A) according to the Joanna Briggs Institute Critical Appraisal tool.

STUDY	Was the sample frame appropriate to address the target population?	Were study participants sampled in an appropriate way?	Was the sample size adequate?	Were the study subjects and the setting described in detail?	Was the data analysis conducted with sufficient coverage of the identified sample?	Were valid methods used for the identification of the condition?	Was the condition measured in a standard, reliable way for all participants?	Was there appropriate statistical analysis?	Was the response rate adequate, and if not, was the low response rate managed appropriately?	Overall appraisal
**Al-Magsoosi M.J.N. et al, 2023 [14]**	U	Y	U	N	Y	Y	U	Y	N	**4/9 high**
**Ali F.A. et al, 2023 [9]**	Y	Y	Y	Y	Y	Y	U	U	U	**6/9 low**
**AlShakhs A. et al, 2022 [15]**	Y	Y	Y	N	Y	Y	U	U	U	**5/9 moderate**
**Ansari S. et al, 2023 [101]**	Y	Y	Y	N	Y	Y	U	U	U	**5/9 moderate**
**Binmadi N.O. et al, 2022 [16]**	Y	Y	Y	Y	Y	Y	Y	Y	N	**8/9 low**
**Chawla J. et al, 2022 [18]**	Y	Y	Y	N	Y	Y	U	U	U	**5/9 moderate**
**El Kady D.M. et al, 2021 [21]**	Y	Y	Y	N	N	Y	U	U	Y	**5/9 moderate**
**El Tantawi M. et al, 2022 [22]**	Y	Y	Y	N	Y	Y	U	U	U	**5/9 moderate**
**Elamrousy W.A.H. et al, 2021 [75]**	Y	Y	Y	N	Y	Y	U	U	U	**5/9 moderate**
**Favia G. et al, 2021 [25]**	Y	Y	Y	Y	Y	Y	Y	Y	N	**8/9 low**

**Favia G. et al, 2023 [77]**	Y	Y	Y	Y	Y	Y	Y	Y	N	**8/9 low**
**Ferdeghini C. et al, 2022 [27]**	Y	Y	Y	N	Y	Y	U	U	U	**5/9 moderate**
**Fernandes T.J. et al, 2023 [28]**	Y	Y	Y	Y	U	Y	Y	Y	N	**7/9 low**
**Fidan V. et al, 2021 [79]**	U	Y	U	N	Y	Y	U	U	N	**3/9 high**
**Flores-Silva F.D. et al, 2021 [29]**	Y	Y	Y	Y	Y	Y	Y	N	N	**7/9 low**
**Ganesan A. et al, 2022 [30]**	Y	Y	Y	Y	Y	Y	Y	Y	N	**8/9 low**
**Haran J.P. et al, 2021 [34]**	U	Y	U	N	U	Y	U	Y	N	**3/9 high**
**Hussain S. et al, 2023 [37]**	U	Y	U	N	Y	Y	U	U	N	**3/9 high**
**Hussain S. et al, 2023 [35]**	U	Y	U	N	Y	Y	U	Y	N	**4/9 high**
**Jain A. et al, 2020 [36]**	U	Y	U	N	Y	Y	U	U	N	**3/9 high**
**Limongelli L. et al, 2023 [84]**	Y	Y	Y	Y	Y	Y	Y	N	N	**7/9 low**
**Martinez F. et al, 2022 [42]**	Y	Y	Y	Y	Y	Y	Y	N	N	**7/9 low**

**Metin N. et al, 2024 [43]**	Y	U	Y	Y	Y	Y	Y	Y	U	**7/9 low**
**Miyazato Y. et al, 2022 [44]**	Y	Y	Y	Y	Y	Y	Y	Y	N	**8/9 low**
**Mohammed F. et al, 2023 [46]**	Y	Y	Y	N	U	Y	Y	Y	Y	**7/9 low**
**Muthyam A.K. et al, 2022 [48]**	Y	Y	U	U	Y	Y	Y	Y	U	**6/9 low**
**Natto Z.S. et al, 2021 [50]**	Y	Y	Y	U	Y	Y	Y	U	U	**6/9 low**
**Nikalje M.R. et al, 2021 [51]**	U	Y	U	N	Y	Y	U	Y	N	**4/9 high**
**Nikalje M.R. et al, 2021 [51]**	U	Y	U	N	Y	Y	U	U	N	**3/9 high**
**Paszynska E. et al, 2023 [53]**	Y	Y	Y	Y	Y	Y	Y	Y	N	**8/9 low**
**Patel D. et al, 2024 [54]**	Y	Y	Y	N	Y	Y	U	U	U	**5/9 moderate**
**Ramasamy K. et al, 2020 [55]**	Y	Y	Y	N	Y	Y	U	U	U	**5/9 moderate**
**Rogn A. et al, 2024 [56]**	Y	Y	U	Y	Y	Y	Y	Y	N	**7/9 low**
**Sheng W.H. et al, 2021 [61]**	Y	Y	Y	Y	Y	Y	Y	N	N	**7/9 low**

**Sørensen A.I.V. et al, 2022 [64]**	Y	Y	Y	N	N	Y	U	U	Y	**5/9 moderate**
**Subramaniam T. et al, 2021 [95]**	Y	Y	Y	Y	Y	Y	U	U	U	**6/9 low**
**Tavakoli F. et al, 2022 [96]**	U	Y	U	N	Y	Y	U	Y	N	**4/9 high**
**Tuter G. et al, 2022 [68]**	Y	Y	Y	Y	Y	Y	Y	N	N	**7/9 low**
**Vijapur M.M. et al, 2022 [113]**	Y	Y	Y	N	Y	Y	U	U	U	**5/9 moderate**

Table S5: Assessment of quality and risk of bias for cross-sectional and observational studies included in the systematic review. Each domain was satisfied (Y), not satisfied (N), unclear (U), or not applicable (N/A) according to the Joanna Briggs Institute Critical Appraisal tool.
